# Supplementary figures and images for: Spiking activity in the visual thalamus is coupled to pupil dynamics across temporal scales
Source: PLoS Biol. 2024 May 14;22(5):e3002614. doi: 10.1371/journal.pbio.3002614 (PMC11093384; doi:10.1371/journal.pbio.3002614)

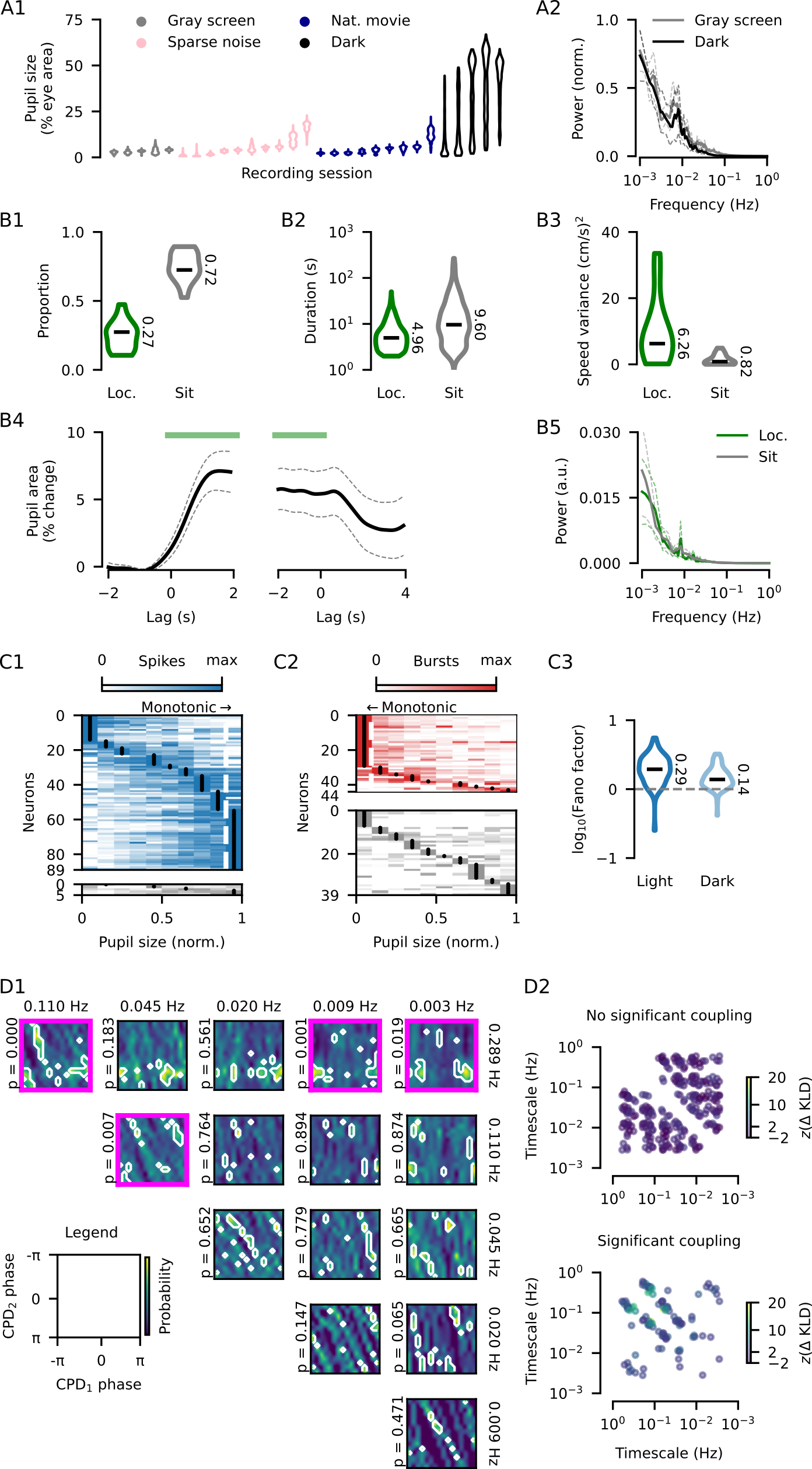

Supplement: S1 Fig — (A1) Pupil size distributions for all recording sessions. Pupil size is expressed as a fraction of the total exposed eye area (see Materials and methods). Data from the gray screen and sparse noise sessions are grouped together for the analyses in Figs 1, 2, 4, S2, S3B, S3C and S4. (A2) Pupil size power spectral density (mean ± SEM, min-max normalized) for gray screen recordings (N = 5) and recordings in darkness (N = 5). (B1) Distribution of proportion of time spent in a locomotion bout (green, see Materials and methods) versus sitting (gray) for N = 14 sessions where locomotion speed was recorded (Horizontal bars: median proportion). (B2) Distribution of locomotion bout and inter-bout-interval lengths. (B3) Distribution of locomotion speed variance during locomotion bouts versus sitting. Whereas locomotion bouts were characterized by larger behavioral variability, the inter-bout-intervals had very low variability in locomotion speed. (B4) Pupil size (mean ± SEM, normalized to the pre-bout pupil area) surrounding locomotion bout onsets (left) and offsets (right). (B5) Pupil size power spectral density (mean ± SEM) for periods of locomotion (green) and quiescence (gray). (C1) Spike counts (min-max normalized) across pupil sizes (min-max normalized) for the dLGN neurons with significant modulation by pupil size (top, one-way ANOVA across 10 pupil size bins, p ≤ 0.05) and without significant modulation (bottom) during recordings performed in darkness. Neurons are sorted by the location of the maximum firing rate (black dots). The majority of significantly modulated neurons (61.8%) had “non-monotonic” modulation profiles, with their maximum firing rates outside of the 90th percentile of pupil size (dashed white line). (C2) Same as (C1) but for bursts of spikes. Neurons are sorted by the location of the maximum burst rate (black dots). The majority of significantly modulate neurons had “monotonic” modulation profiles, with their maximum burst rates in the 10th percentil [file pbio.3002614.s001.tiff]

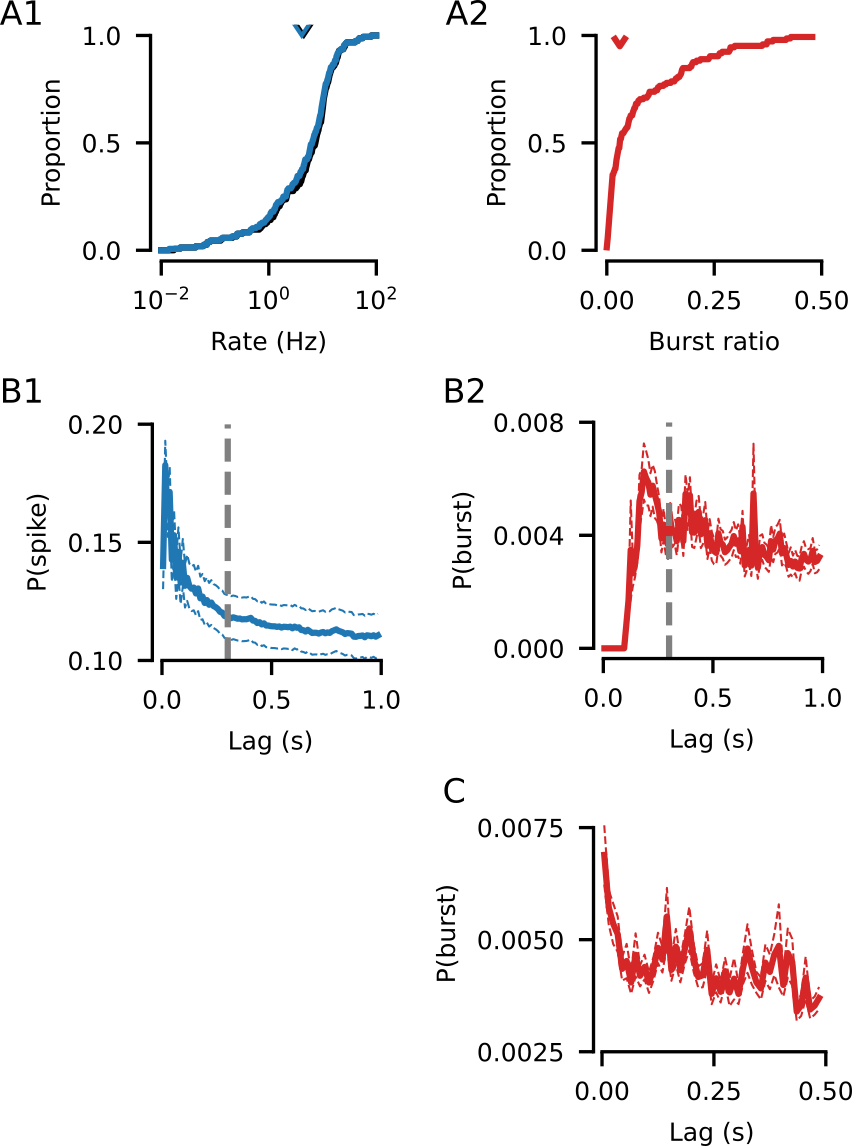

Supplement: S2 Fig — (A1) Distribution of firing rates (black) for all neurons recorded in spontaneous or sparse noise sessions (mean = 4.49 spk/s, N = 156). Blue: The firing rate distribution considering only tonic spikes (mean = 4.11 spk/s), showing that overall firing rates were primarily determined by tonic spiking. Neurons with a firing rate <0.01 spk/s were excluded from all analyses. (A2) Distribution of burst ratios (number of spikes assigned to a burst/total number of spikes, median = 3.0%). Bursts were detected in 92.9% of neurons (145/156). (B1) Mean auto-correlogram of tonic spiking (mean ± SEM). The dashed line at 300 ms indicates the bin-width used to generate shuffled spike trains used to test phase coupling significance (see Materials and methods). (B2) Mean auto-correlogram of bursting (mean ± SEM). Note that the peaks are spaced apart by approximately 200 ms, indicating that approximately 5 Hz rhythmic bursting was present (see also Nestvogel and colleagues). (C) Mean burst cross-correlogram (mean ± SEM) for all simultaneously recorded pairs of neurons (N = 1,185 pairs). The peaks at zero and approximately 200 ms indicate that neurons tend to burst synchronously and rhythmically. (TIFF) [file pbio.3002614.s002.tiff]

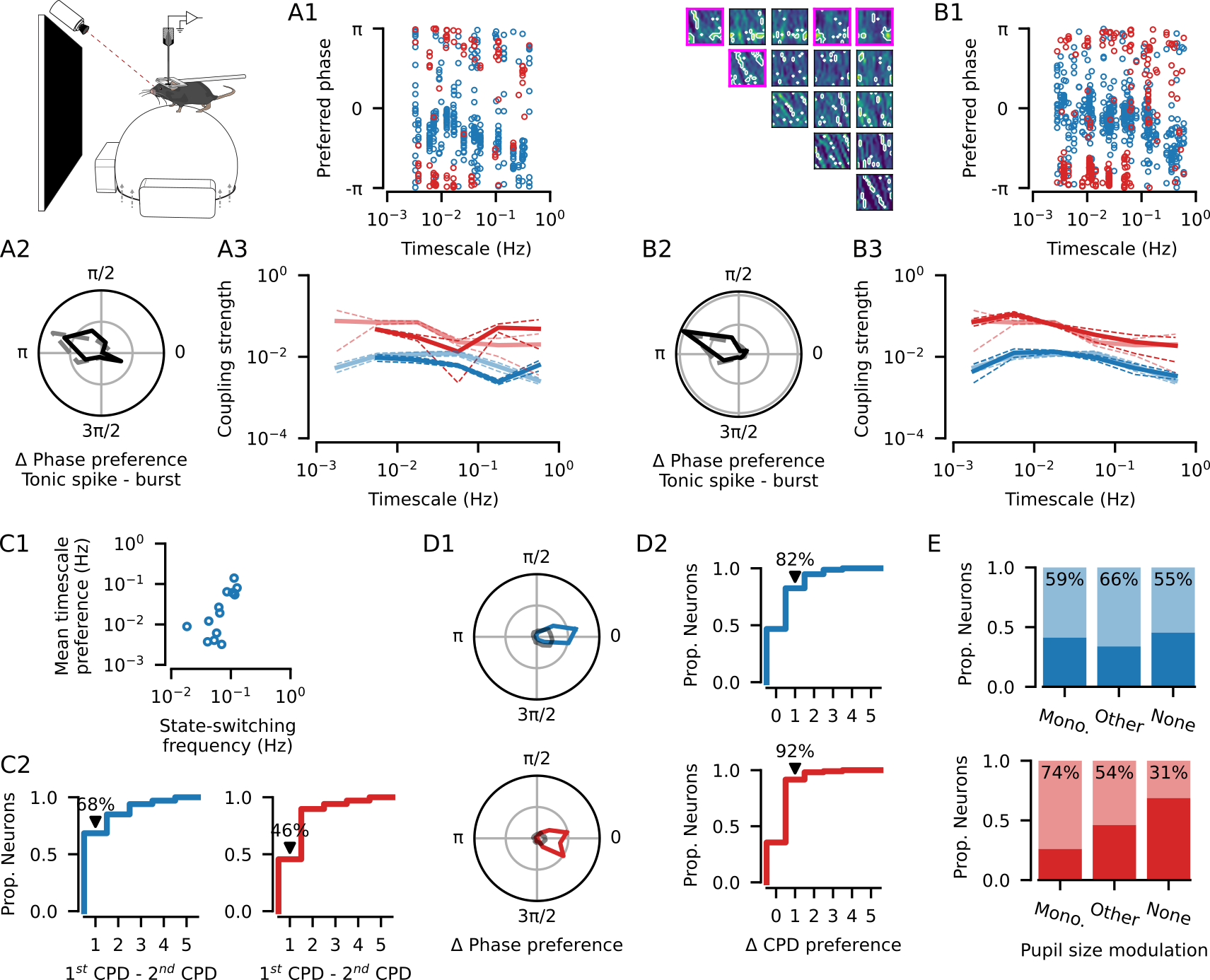

Supplement: S3 Fig — (A) Phase coupling analysis relating dLGN spiking to CPDs for recordings performed in darkness. (A1) Preferred coupling phase of tonic spikes (blue) and bursts (red) for all neuron-CPD pairs with significant coupling. Significant tonic spike coupling was observed in 96.8% of neurons (91/94; mean = 3.4 CPDs per neuron) and burst coupling in 64.8% of neurons (35/54; mean = 1.9 CPDs per neuron). (A2) Distribution of the preferred phase differences between tonic spiking and bursting for neuron-CPD pairs with significant coupling of both spike types (mean = 2.5; N = 88 neuron-CPD pairs; V-test for non-uniformity and a mean of π: V = 25.8, p = 5.1×10^−5^; grid lines indicate proportion of 0.25). While the mean phase difference is similar to Fig 2D (dashed gray line), we also note that in darkness a small proportion of neurons appear to have a spiking pattern where tonic spikes immediately follow bursts without a phase delay (Δ phase preference ~2π). (A3) Coupling strengths (solid lines: median, dashed lines: bootstrapped SE of the median) measured in darkness (bold lines) or in an illuminated environment (faded lines; Fig 2E) for tonic spiking (dark median = 0.0058, N = 321 neuron-CPD pairs vs. illuminated median = 0.0081, N = 682 neuron-CPD pairs; Mann–Whitney U test: U = 1.2×10^5^, p = 2.9×10^−3^) and bursting (dark median = 0.0386, N = 100 neuron-CPD pairs vs. illuminated median = 0.0528, N = 320 neuron-CPD pairs; Mann–Whitney U test: U = 1.8×10^4^, p = 0.05). (B) Phase coupling analysis performed on the same recordings as in Fig 2, but excluding spikes that occurred during periods of phase coupling between CPDs (S1D Fig; see Materials and methods). (B1) Preferred coupling phase of tonic spikes (blue) and bursts (red) for all neuron-CPD pairs with significant coupling. Significant tonic spike coupling was observed in 98.6% of neurons (146/148; mean = 3.8 CPDs per neuron) and burst coupling in 74.2% of neurons (89/120; mean = 2.1 CPDs per neuron). (B2) Distribution of the prefe [file pbio.3002614.s003.tiff]

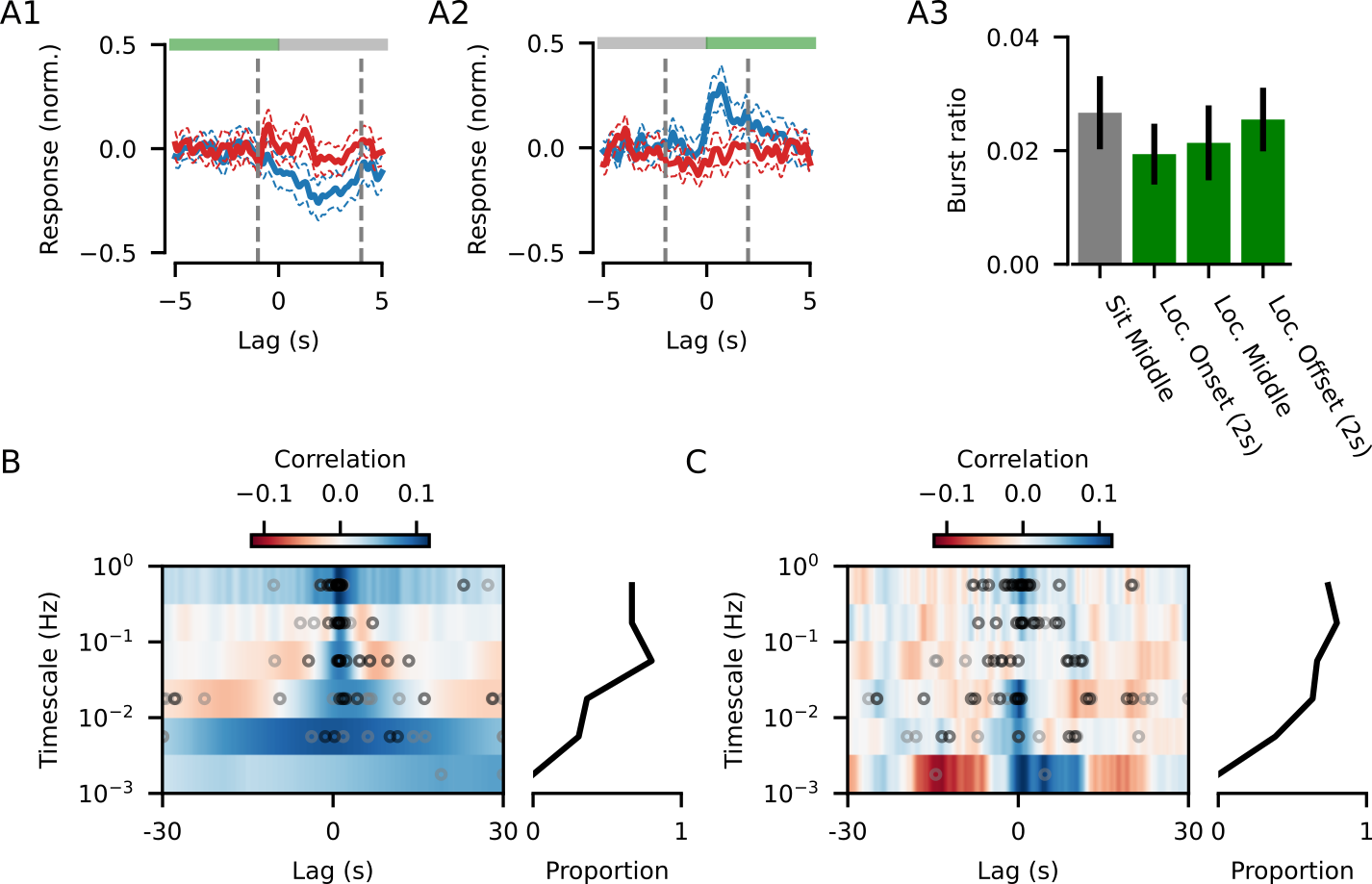

Supplement: S4 Fig — (A1) Firing rate responses to the offset of locomotion bouts (mean ± SEM, relative to the baseline from −5 to −3 s and min-max normalized for each neuron) for tonic spiking (blue; N = 111/121 neurons with significant tonic spiking modulation) and bursting (red; N = 98/121 neurons with significant bursting modulation). Vertical lines denote the period surrounding the offset of locomotion bouts during which the mean firing rate deviates from baseline (−1 s to 4 s), and therefore spiking activity during this transition period was excluded for the analysis in Fig 4A, in addition to all activity during locomotion bouts. (A2) Same as (A1) but for the onset of a locomotion bout (N = 108/121 neurons with significant tonic spiking modulation; N = 96/121 neurons with significant bursting modulation). Vertical lines denote the transition period (−2 s to 2 s) surrounding the onset of a bout from which spiking activity was excluded for the analysis in Fig 4B, in addition to all activity during quiescence. (A3) Median burst ratio (number of burst spikes/total number of spikes, error bars: bootstrapped SE of the median) across quiescence (gray, excluding 2 s prior to and following locomotion bouts) and locomotion (green, split for the first 2 s of bouts, the middle portion of bouts, and the final 2 s of bouts) showing burst spikes are less prevalent, but not absent, during locomotion (Friedman chi-squared test: Q = 22.0, p = 6.7×10^−5^). (B) Left: Mean cross-correlation between CPDs and locomotion speed, grouped by the timescale of the CPD (black dots: location of the peak correlation for each CPD with a significant correlation; gray dots: location of the peak correlation for each component without a significant correlation). Correlation significance was determined by comparing the maximum value of the cross-correlation to a null-distribution obtained by correlating the CPD with locomotion speed traces taken from different recording sessions (see Materials and methods). To eliminate [file pbio.3002614.s004.tiff]

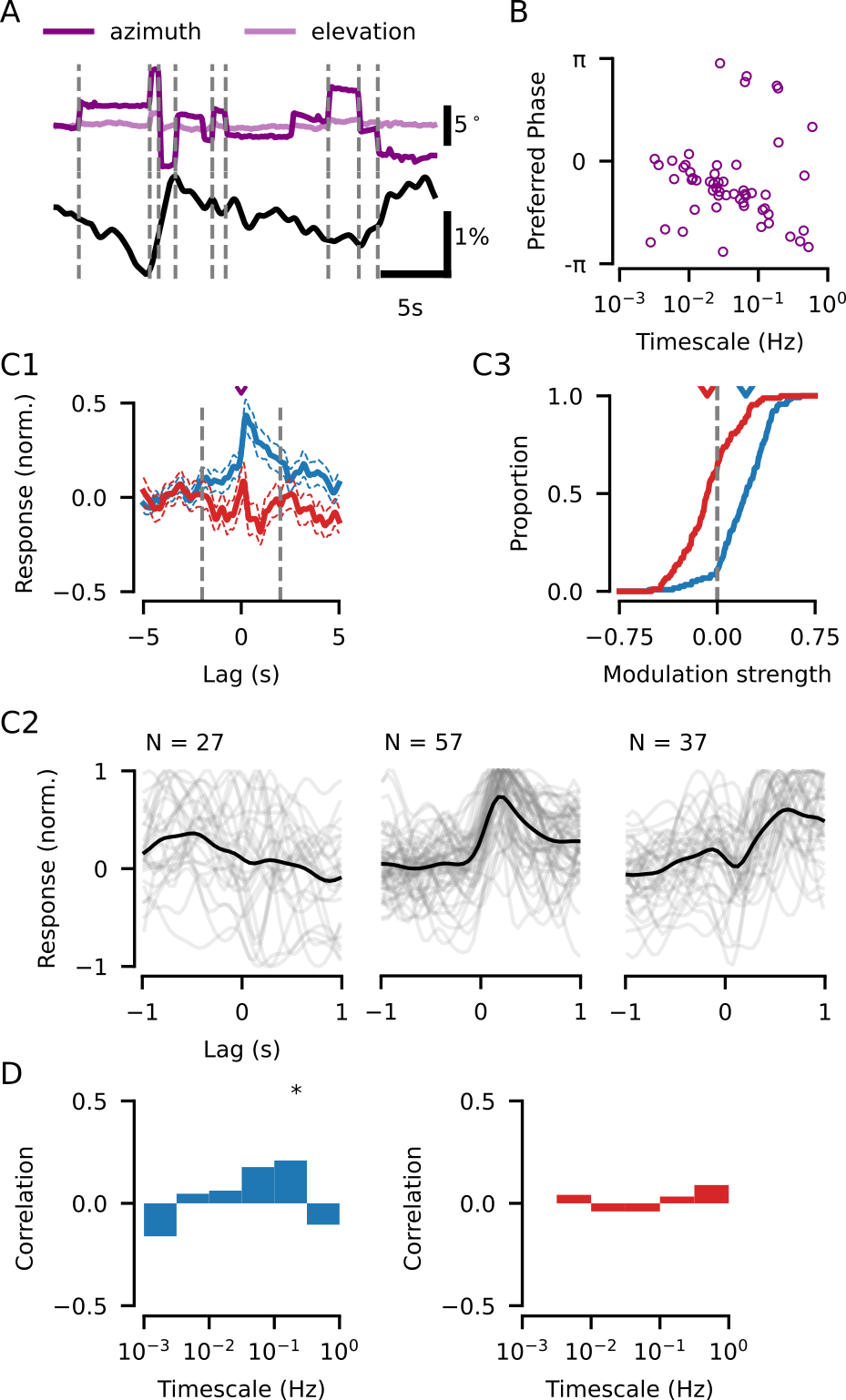

Supplement: S5 Fig — (A) Example eye position (top) and pupil size (bottom) traces. Detected saccades are marked by the dashed lines. (B) This coupling of saccades to pupil dynamics was verified using the same phase coupling analysis as in Fig 2, relating saccades to each of the CPD. Shown here are the preferred phases at which saccades occur, for CPDs across various timescales (significant saccade coupling to at least 1 CPD was observed in 15/15 recording sessions). Across all timescales, saccades tend to occur during pupil dilations, similar to tonic spikes (Fig 2C). (C1) Firing rate responses to saccades (mean ± SEM, relative to the baseline from −5 to −3 s and min-max normalized for each neuron) for tonic spiking (blue; N = 118/121 neurons with significant tonic spiking modulation) and bursting (red; N = 88/121 neurons with significant bursting modulation). Vertical lines denote the period surrounding saccades during which the mean firing rate deviates from baseline (−2 s to 2 s); therefore, spiking activity during this transition period was excluded for the analysis in Fig 4C. (C2) Neurons in the dLGN have diverse saccade-triggered tonic spiking responses. Clustering the normalized peri-saccadic responses (see Materials and methods) revealed at least 2 distinct response types, in addition to a minimally responsive/mixed cluster (left). The first responsive cluster (middle) had a transient increase in firing tightly locked to saccade onsets. The second responsive cluster (right) was characterized by gradually increased firing rates prior to saccade onset, with a brief suppression immediately following saccade onset, before returning to a sustained facilitation. (C3) Despite the diversity in responses, the peri-saccadic period (−2 to 2 s) was characterized by an overall increase in tonic spiking rates. The modulation was quantified by taking the area under the normalized saccadic response curve in a window spanning −2 to 2 s for tonic spiking (median = 0.2; Wilcoxon rank-sum test: W [file pbio.3002614.s005.tiff]

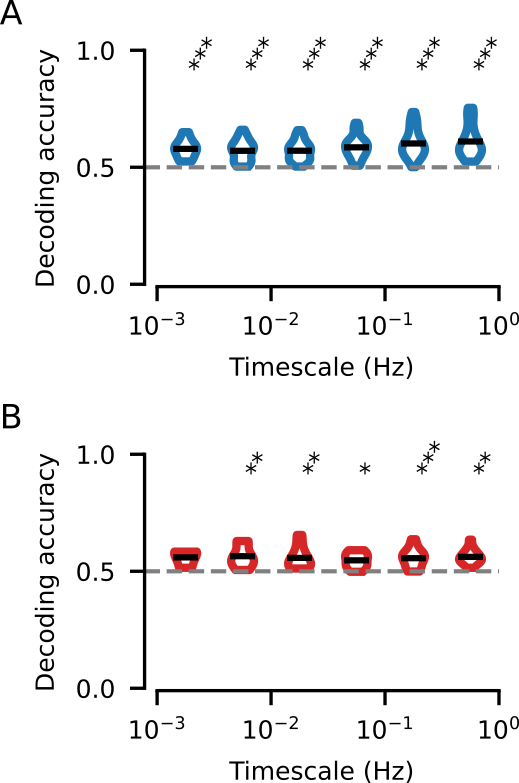

Supplement: S6 Fig — (A) Distributions of CPD phase decoding accuracy using tonic spiking (N = 273 neuron-CPD pairs; dashed line: chance-level performance). For each neuron with significant tonic spike-CPD coupling, a support-vector classifier was trained to select between 2 phase bins, one of which was centered around the preferred phase of tonic spike coupling, the other was centered 180° opposite. Decoding accuracy was cross-validated using 5 training-test splits. The distribution of decoding accuracy was significantly greater than chance for all timescales (Wilcoxon rank-sum test for each timescale, p-value denoted by asterisks, * for p ≤ 0.05, ** for p ≤ 0.01, *** for p ≤ 0.001). (B) Same as (A), but for CPD phase decoding using bursting. For each neuron with significant burst-CPD coupling, a support-vector classifier was trained using bursting activity, and the phase bins were centered around the preferred phase of bursting. (TIFF) [file pbio.3002614.s006.tiff]
