# Supplementary material for: Spiking activity in the visual thalamus is coupled to pupil dynamics across temporal scales
Source: PLoS Biol. 2024 May 14;22(5):e3002614. doi: 10.1371/journal.pbio.3002614 (PMC11093384; doi:10.1371/journal.pbio.3002614)
Supplement: Attachment — Submitted filename: Crombie21_response.pdf [file pbio.3002614.s007.pdf]

Dear Dr. Smith, dear editorial team, dear reviewers,

We gratefully appreciate the opportunity to revise and resubmit our manuscript entitled "Spiking activity in the visual thalamus is coupled to pupil dynamics across temporal scales" (formerly, "Modulation of dLGN firing mode across multiple timescales is predicted by pupil size dynamics"), which was reviewed in PLoS Biology as a Short Report in the summer of 2021. We found both the editor's notes and the reviewers' comments very insightful and constructive. After careful consideration of their feedback, we have chosen to invest additional time and effort to add more data and conduct new analyses, to rule out alternative interpretations of our data and further support the validity of our conclusions. In addition, we have overhauled the text, to enhance its clarity and accessibility to a broader readership. We firmly believe that these changes have now made the manuscript a strong candidate for publication in PLoS Biology. The most prominent changes are summarized here:

1) In order to address the concerns raised by all three reviewers and better integrate our findings with the existing literature, we have made several modifications to the manuscript. Specifically, we now provide a more comprehensive quantification of the animals' behavioral states that we are able to sample in our recordings. Additionally, we have provided a detailed description of how pupil size *per se* is related to spiking in the dorsolateral geniculate nucleus (dLGN). The new analyses, presented in Figure 1, show that while pupil size *per se* can be used as a marker for internal state, the relationship between pupil size and spiking activity varies across neurons, and may not fully capture the complexity of internal states in the dLGN. We believe that adding this information to the manuscript, accompanied by the updated figures, has allowed us to better align our results with the existing literature, and provides a strong justification for the analyses employed in our study.

2) We have repeated our analyses for a new dataset that includes more recordings with a purely isoluminant gray screen. For this new data, we were able to replicate all our results, confirming their robustness (Figures 1-4). This new data set helps us to address concerns raised by Reviewer 3, allowing us to show that the data from spontaneous (gray screen) and sparse noise conditions yield identical conclusions with respect to phase coupling between pupil dynamics and dLGN spiking (Figure R6 on page 10). Based on these empirical considerations, as well as our assumption that the sparse noise stimulus does not induce pupil size changes, we conclude that it is a valid approach to pool data across these conditions.

3) In response to a question by reviewer 2 regarding the sources of the coupling between dLGN spiking activity and pupil changes, we have also added new data from recordings performed in darkness. We applied the same analyses on these dark-recorded data, for which the results are presented in Figure S1C and Figure S2. This additional data shows that our main conclusions are not driven by changes in retinal irradiance, which might occur as the pupil dilates and constricts. This additional evidence thus supports the interpretation that the modulations we observed were driven by internal factors, rather than external light conditions.

As advised by the editor, we have carefully re-written the manuscript to place a stronger emphasis on the results and their biological relevance. In doing so, we have eliminated abbreviations and technical language, and removed descriptions of the methodology from the main text. We believe that these revisions and further changes to the figures have greatly enhanced the readability and broadened the appeal of the manuscript.

In addition to the changes suggested by the reviewers and the editor, we have made several changes on our own accord:

1) The method we chose to decompose pupil dynamics offers the advantage of yielding frequency modulated components that effectively capture non-oscillatory pupil dynamics. A potential downside, however, is that the relationship between these components is not known *a priori* and must be measured. Potential phase relationships between components would constitute confounds when assessing phase coupling to multiple components, since the observed coupling with multiple components may be influenced by relationships among the components themselves, rather than to the spiking activity. To address this concern, we developed a statistical test to determine if and when these periods of phase synchrony occur (Figure S1D). By removing the contribution of spiking during these periods, we found that the phase coupling remains largely unchanged (Figure S3B). Thus we conclude that dLGN spiking is genuinely modulated at multiple temporal scales, and that the observed multi-component coupling is not an artifact stemming from relationships between the components of pupil dynamics.

2) We have delved deeper into the consequences of phase coupling between pupil dynamics and dLGN spiking for stimulus encoding. In a new set of analyses (Figure 5 and S6) we show that coupling to multi-scale pupil

dynamics persists during naturalistic stimulus viewing, and that this coupling contributes to differences in stimulus encoding across different phases of pupil dynamics. We believe that this new Figure further enhances the impact of our findings, by showing that the modulations we uncovered have substantial effects on how the visual system encodes information dynamically throughout wakefulness.

Below, we detail the steps we have taken to address each of the reviewers' comments (in blue). We hope that we have been able to address all of your concerns.

Reviewer 1

This study examines the state dependency of thalamic burst and tonic firing modes. The authors find that bursts are more frequently seen with pupillary constriction while tonic firing is observed more frequently with pupil dilation. There are also some dominant frequencies at which these pupillary dynamics occur, but the frequency distribution is very broad. This study is relevant to the understanding of the state dependence of thalamocortical interactions and will be valued by the community interested in this topic. I have several suggestions for improving the manuscript.

We thank the reviewer for appreciating our study and for the helpful comments.

1) Line 33, the authors state "a hyperpolarization activated Ca2+ current". This Ca2+ current is not activated by hyperpolarization. It is activated by depolarization. The hyperpolarization removes the inactivation of the current (de-inactivation), allowing low threshold Ca2+ spikes to occur in response to a depolarizing event such as an EPSP or the withdrawal of an IPSP or the activation of the h-current. Perhaps stating "a Ca2+ current that is dependent upon hyperpolarization for its "activation" would be more appropriate.

We thank the reviewer for identifying this issue. We have changed the wording to more accurately reflect the underlying processes. The new sentence reads:

> *Burst firing in the thalamus is characterized by a high frequency discharge of action potentials after sustained hyperpolarization, and it relies on the activation of low-threshold, transient calcium (T) channels. In contrast, tonic firing occurs when the membrane potential is relatively depolarized, and T-type calcium channels are inactivated (Sherman, 2001).*

2) Line 45 - The statement "could occur independently of locomotion" is misleading. This could mean practically anything, including that you saw one burst that occurred during locomotion while all the others did not. It would be more informative to relate the statistical distribution of burst and tonic firing probabilities to distinct locomotion states (e.g. beginning, middle, end, quiescence, etc.).

We agree that the previous statement was ambiguous, and we have revised it to more precisely reflect our results. This section of our abstract now reads: *"This phase-coupling could not be explained by... transitions between overt locomotion and quiescence, ..."*. As suggested by the reviewer, we conducted an analysis to quantify the degree of burst firing during distinct locomotor states (Figure R1 on page 3). These results align with previous studies [2, 6] and demonstrate that bursting is more prevalent during quiescence than locomotion. However, it is important to note that bursting still occurs during locomotion. Since the quantification of burst ratios in the dLGN during locomotion have already been published in Niell and Stryker [6] and Erisken et al. [2], we have decided not to include this figure in our manuscript. Nevertheless, we are open to moving these analyses to the supplementary material if the reviewer finds them valuable.

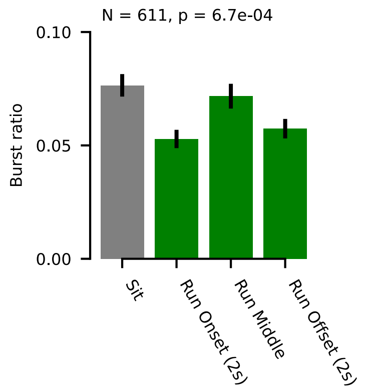

Figure R1 Burst rates (proportion of all spikes that are part of a burst) for various behavioral states. Error bars represent the SEM, p-value results from a one-way ANOVA across states.

3) Figure 1. The max pupil diameter appears rather small - perhaps there was a lot of light in the recording setup? I am not sure if that limits the ability of the authors to see the full dynamic range of pupil dynamics, perhaps through a floor effect, but hopefully not. In the future, less light in the chamber may help record larger pupillary changes.

We thank the reviewer for this thoughtful observation. Our original intention was to assess arousal-related modulations of dLGN activity under typical experimental stimulus viewing conditions, i.e. when there is some light from the monitor in the recording chamber. However, the reviewer's observation prompted us to consider two important questions: 1) whether these viewing conditions limit the pupil's ability to reflect internal changes, and 2) whether luminance-related mechanisms, such as changes in retinal irradiance for different pupil sizes, could account for our observed results, an issue also raised by Reviewer 2. To address these questions we have added data recorded in darkness to the new version of our manuscript. We find that, while the range of pupil sizes is increased in darkness compared to the gray-screen condition (Figure S1A), the pupil dynamics are preserved (Figure R2). Poignantly, our main conclusions from Figure 1 and 2 obtained when there is light present in the recording chamber were also found in darkness (Figure S1C and S3A), supporting our interpretation that our results are due to internal factors, and not the illumination levels during the recording.

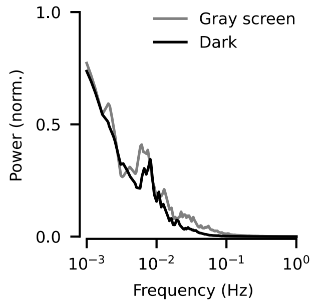

Figure R2 Mean power spectral density of the pupil size signal – derived via the Hilbert-Huang Transform [4], as in our manuscript – for recordings performed with a gray screen and in darkness

4) Personally, I found the manuscript difficult to read and understand. Part of the difficulty arises from the frequent use of abbreviations such as PS, IMF, etc. Another part is the non-intuitive way in which the data was analyzed. Why not plot data in a more intuitive way to start and then proceed to the current analysis? For example, plotting the rate of occurrence of bursts and tonic firing for different pupil diameters (with a delay, see below) would be intuitive. Doing the same for the rate of constriction and dilation (e.g. derivative of pupil diameter) would also be intuitive. Perhaps plotting the rate of occurrence of bursts and tonic firing versus running speed would also be interesting. Or the rate of tonic firing and bursting against the timing of onset and offset of running or pupil dilation/constriction - this would be very informative. The way the current manuscript is written, understanding the

results requires a deep understanding of "IMF" and that reduces the impact of the paper considerably, in my opinion.

We appreciate the reviewer's feedback, and in response we have made significant improvements to the readability of our manuscript. To address the concerns raised, we have provided a more extensive motivation for the choice of analysis and have extensively revised the technical language used. As suggested, we have removed abbreviations such as PS and IMF, and replaced them with more intuitive terms.

In response to the reviewer's suggestion, we have added several new analyses at the beginning of the results section. These analyses investigate how firing rates in the dLGN change in relation to pupil size (Figure 1C), which provides a more conventional approach for understanding arousal-related changes. Similarly, we have explored the derivative of pupil size as an alternative measure, but have not included the data in the manuscript as the conclusions are largely equivalent to those obtained with pupil size *per se*, while the derivative is generally a worse predictor of spiking activity (Figure R3 on page 4).

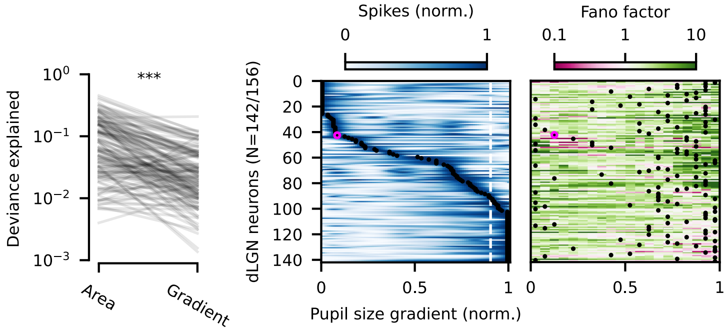

Figure R3 Left: deviance explained by PoissonGAM model for each unit using pupil area or gradient as regressors (Wilcoxon rank-sum test p $\leq$ 0.001). Pupil size is a better predictor of firing rates than its temporal derivative. Middle: model fits to the pupil gradient (min-max normalized), arranged by the location of the peak firing rate. Right: Fano factor across pupil gradient values, with the same sorting as in the middle panel. The example neuron from Figure 1 is highlighted in magenta.

Lastly, we have taken efforts to provide more intuitive descriptions of what the components of pupil dynamics represent when introducing the method (results text associated with Figure 1D) and have further discussed the potential biological and psychological factors that may underlie these dynamics in the discussion section. Overall, we believe that these revisions allow for a better understanding of our research findings.

5) Pupil dynamics are slow and delayed, owing to the smooth muscle nature and g-protein mediated neurotransmitter signaling. The activity in the parasympathetic and sympathetic axons is perhaps more accurate to rapid changes in state in the animal, but these are not readily available. The pupil is delayed by approximately 0.5 second from an increase in discharge in the sympathetic nervous system, for example. And the response is smoothed out in time over a prolonged period. How does this low frequency filtering by the pupillary muscles affect the interpretation of the results? Have the authors taken into account the delay and slow dynamics in their analysis? What if the bursting and/or tonic firing in the thalamus was also smoothed and delayed to a similar extent as the pupil - would that change the results?

We thank the reviewer for a very thoughtful comment. We have interpreted these questions, together with comment 8, to be referring to the fact that a signal (here: the internal state change), when subject to a filter such as a temporal delay or low-pass filtering (here: transmission across synapses with potentially slower receptors, and the response time of the pupillary muscles, for example), will undergo phase delay, whereby the different frequency components of the signal will experience a relative phase shift. This would manifest in a change in the apparent phase relationship of another signal (here: spiking in the dLGN) with the various frequency components of the filtered signal (here: the pupil size).

Therefore, in Figure 2C, the observed phase shift across frequencies could indeed be influenced by the phase response of the pupil size filter rather than reflecting a genuine difference in the dLGN response to internal state changes occurring across different timescales. To investigate this further, we assumed a linear phase response and

calculated the temporal delay using the slope of a line fitted to the phase tuning of e.g. tonic spikes. This analysis yielded a biologically plausible delay of 0.3 seconds Figure R4 on page 5. We note, however, that the data can be equally well captured by a linear fit to log-frequency, contradicting our simplifying assumption that the phase response is linear. This approach further assumes that the spiking response occurs at the same phase of the internal signal for all of its frequency components, which seems unlikely given the myriad direct and indirect mechanisms in which arousal-related neurotransmitters can influence dLGN neurons. Accordingly, as the reviewer rightly points out, the spiking activity itself could be a filtered version of the signal causing the internal state change. This further complicates the interpretation of the phase-frequency relationship between the spiking and the internal signal.

We chose not to emphasize the phase-frequency relationship in the manuscript due to these challenges in interpretation, although we agree with the reviewer that this is a salient feature of the results, and will happily include the above considerations in the manuscript if the reviewer thinks they will be of general interest. Lastly, we want to emphasize that these considerations do not affect our main results: 1) the modulation of dLGN spiking at multiple temporal scales, and 2) the occurrence of burst and tonic firing at opposing phases within each temporal scale.

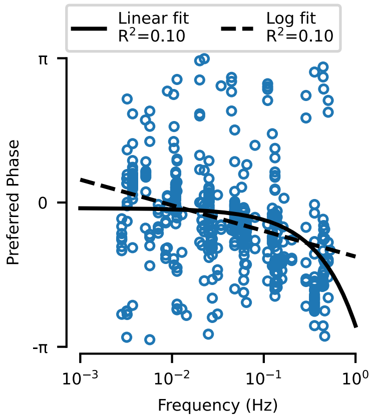

Figure R4 Linear and logarithmic fits to the phase-frequency relationship of tonic spike phase tuning. Note the log-scaling of the x-axis.

6) In figure 2C, what is p=0.000 mean? Please put p<0.001 or such.

To assess the significance of phase coupling, we use permutation tests with 1000 shuffles, allowing us to determine p-values empirically with a precision of three decimal places. Hence, when we report $p = 0.000$, it means that none of the shuffled samples had a value more extreme than the actual test sample. We apologize for any confusion caused and have now provided clarification in the Methods section.

7) In figure 2C, the bursts appear to be well tuned to phase, while the tonic discharge is just barely tuned. This should be pointed out if it is a general observation.

Indeed, this is a general observation visible across timescales (Figure 2E) and is one of the main signatures of the phase tuning we observe. We now include our interpretations of this point in the text associated with Figure 2E.

8) What effect does the intrinsic dynamics of the pupil (delay and low frequency smoothing) have on the plot in Figure 2D? I suspect that several features of the plot are the result of the pupillary dynamics as opposed to the activity of the sympathetic/parasympathetic pathways.

We again thank the reviewer for these insights, and hope that our response to point 5) covers the question raised here.

9) Line 154. Reimer et al. specifically looked at constriction/dilation as the best indicator of state. This should be mentioned.

Indeed, previous studies by Reimer et al. [7, 8] in the primary visual cortex of mice investigated the phases of pupil dilation and contraction rather than pupil size. In the revised manuscript, we have made sure to explicitly refer to their work in the results section before introducing our method, and further in the discussion to acknowledge the foundation upon which our study is built. However, it is important to note that our methodology extends considerably beyond this initial starting point, emphasizing the coupling of spikes to multiple nested temporal frequencies, and identifying a difference in phase coupling for bursts and tonic spikes at a pre-cortical level.

10) The data in the present paper concerns only a subset of the data from a fully behaving animal. There is a preponderance of data from animals that are undergoing frequent changes in pupil size. Long bouts of running, in which the pupil are strongly dilated for prolonged periods of time, seem to be missing. Periods when the animal is in REM sleep and the pupils are pin-point are also likely missing. These lack of prolonged periods of a particular state may lead to conclusions that are not appropriate - such as only pupil dilation and constriction, and not pupil size, indicate state or burst/tonic discharge in the thalamus. The authors should be more circumspect about their results in this regard.

We agree with the reviewer that sampling of behavioral states during experiments such as ours may be biased, as the experimenter has limited control over the internal state of the animal. To address this concern, we have made several additions to the manuscript. Firstly, we have included a quantification of the distribution of pupil sizes for all recordings (Figure S1A), and of the time spent running versus sitting across recordings (Figure S1B1 and S1B2) so that the reader may better assess the behavioral states that we were able to sample. Secondly, we also compared the pupil sizes (Figure S1B3) and dynamics (Figure R5) during bouts of locomotion and quiescence, and of the correlation of pupil size dynamics and speed during locomotion bouts (Figure S4C). Our analysis revealed that, although the dynamic range of the pupil was smaller during locomotion (Figure S1B2), the dynamics between the two states were similar (Figure R5). These results emphasize that in the behavioral states we sample the pupil is constantly dynamic, and rarely fixed at a particular size for a prolonged period of time, supporting our focus on dynamics rather than size *per se*.

Furthermore, we have now clarified when summarizing our results in the discussion that our results and interpretations are reflect the waking state only. Indeed, sleep-like periods often involved partial or complete eyelid closure, rendering pupil tracking from video recordings impossible. Therefore, we had to exclude many such time periods when the pupil was at its smallest, which likely included REM and other sleep-related states. We now explicitly mention this restriction to ensure a clear understanding of the scope of our study.

11) The statement on line 200 "We further corroborated that pupil size dynamics and overt locomotion are independent" is clearly wrong or misleading since pupil dynamics are highly related to state of the animal, which is highly related to movements of the face and body, including locomotion. Perhaps what the authors mean is that during the relatively stable periods of locomotion or quiescence, there is a similar relationship between LGNd firing and pupil dilation/constriction (although again, this needs to be interpreted on top of the reduced rate of bursting during high arousal). Carefully rewriting the text so that the reader is not mislead is warranted.

We thank the reviewer for catching this error, it is indeed not what we intended to say. We have corrected the sentence and rewritten the section as suggested.

In general, the manuscript is a welcome addition to the understanding of the state-dependence of activity in the thalamus. To increase the effectiveness of the manuscript, I recommend a more clear analysis of the behavioral and activity variables measured and their relationship to tonic firing/bursting and clear, well balanced, presentation of these relationships.

We thank the reviewer for the positive overall assessment and the constructive suggestions for improving the effectiveness of the manuscript.

Reviewer 2

This study by Crombie and colleagues in the Busse laboratory explores the relationship between pupil size dynamics and neural activity in the LGN of the mouse. Previous work on this topic has been focused on absolute pupil size as an indirect measure of arousal levels, whereas the current study emphasizes a multi-scale analysis of pupil size dynamics. The authors report that pupil size changes occur in an "oscillatory" manner over several temporal scales, and that phases of pupil contraction are associated with burst activity in the LGN whereas phases of pupil dilation are associated with tonic spiking in the LGN. The authors interpret their results as aligning with the broader view that burst activity is most frequent when arousal levels are low and least frequent when arousal levels are high. The study has the potential to make a valuable contribution to the literature; however, there are several aspects of the analysis that raise concern and the interpretation of the overall findings are seemingly unjustified.

We thank the reviewer for appreciating the potential impact of our study, and for the helpful comments addressed below.

Major comments

1) A major finding reported in this study is absolute pupil size is not associated with preferences for burst vs. tonic activity, but rather it is the phase of the pupil dynamics with respect to the IMFs that are reported to be the feature associated with burst vs. tonic activity. With this approach, the link to arousal is seemingly broken. Without the link between absolute pupil size and arousal, one struggles to see the relationship between the current work and behavioral state, and the authors do not provide evidence to establish a link. Further, their own data shows an unchanging relationship between phase and LGN activity across behavioral state.

We would like to point out that it is already common practice to link the dynamic aspects of the pupil, rather than absolute pupil size, to arousal-related cognitive [1, 3, 9] and neurophysiological [7, 8] variables. We acknowledge that pupil size *per se* is still a valuable measure, and have now included an initial analysis demonstrating that pupil size can undoubtedly reflect spiking activity in the dLGN for a large number of neurons (Figure 1C). However, we also use this analysis to show that there may be shortcomings to only using pupil size as a measure of internal state dynamics (Figure 1C2). This serves as a motivation for the subsequent analyses we present. We have now clarified in the first results section and discussion that our decomposition and phase coupling approach is not in contradiction to using pupil size. In fact, tuning with a phase of 0 to a high-power component likely corresponds to pupil size tuning. One of the motivating factors for our work is the understanding that internal and behavioral states are not unidimensional [5]. Therefore, our approach aims to capture these states in greater detail, taking a step towards a more comprehensive assessment.

2) An alternative explanation that the authors should consider is the possibility that stochastic pupil size dynamics are triggering luminance gain mechanisms, which can be quite fast, occur over a large dynamic range, and would be associated with a non-stable response mode. Importantly, this could explain why it is the change in pupil size and not absolute pupil size that matters. This explanation is not provided as being the correct, but rather to illustrate that the data as presented are very difficult to interpret and to assign a biological meaning.

We appreciate the reviewer's valid concern, and in order to address the possibility that luminance gain mechanisms might contribute to our findings, we now include data where dLGN spiking and pupil size were recorded in darkness. This allowed us to eliminate potential influences of luminance-related mechanisms. In our analysis of the data obtained under darkness conditions, we have found that the general pattern of results remain consistent. Specifically, we observe that a similar proportion of neurons show significant coupling to pupil dynamics in darkness (Figure S1C1), with a similar diversity of relationships to pupil size (Figure S1C1). Moreover, we found that firing rate variability was higher than expected (Figure S1C2), similar to the findings obtained under illuminated conditions. Critically, we also repeated our phase tuning analysis for these recordings, and in Figure S3A we observe

that neurons have similar phase coupling characteristics in darkness.

3) Although eye movements are less frequent in rodents than in carnivores or primates, they still occur. The authors should determine whether or not eye movements are related to pupil size dynamics, and if so, whether or not they are confounding the analysis of burst/tonic activity shifts. This is important, as saccadic suppression may prime burst activity separate from arousal related priming.

To address this intriguing possibility, we detected saccades and performed a phase coupling analysis relating saccades to the components of pupil size dynamics (as we did for spiking). We found that saccades tended to occur at phases corresponding to dilation, with a pattern very similar to tonic spiking, i.e. anti-phase with respect to bursting (Figure S5B). We next computed the saccade-triggered averaging tonic spiking for each neuron and found that, despite the presence of some neurons that respond with a brief decrease in tonic spiking (Figure S5C2, right), all neurons were overall positively modulated by saccades. Although there may be a very slight increase in bursting around the time of saccades (Figure R5 on page 8), it is overall unlikely that saccadic suppression contributes meaningfully to the burst phase coupling we observed. We thank the reviewer for this suggestion, which brought about a welcome contribution to our manuscript.

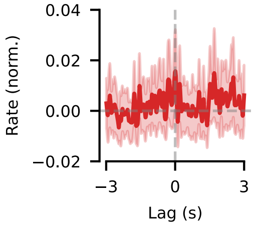

Figure R5 Mean saccade-triggered burst rate across all neurons. Shaded region represents the SEM. Note the greatly reduced y-axis scale compared to Figure S5C1.

4) The term ”oscillation” to describe the pupil size dynamics measured in this study is arguably inappropriate. The range of frequencies (∼0.01-1.0 Hz) over which power is notable is exceptionally broad. The use of the EMD analysis simply allowed the authors to measure power at these frequencies, not to establish significant oscillations at these frequencies. A better term would seem to be ”stochastic”. Vocabulary aside, the authors should include a discussion of what might be driving these events and their biological significance.

The reviewer is correct: the components obtained from EMD can be frequency-modulated and are therefore not strictly “oscillations”. In response, we have removed the term “oscillations” throughout the manuscript. As suggested by the reviewer, we have also expanded our discussion regarding the potential factors driving the components and their biological significance.

5) For Supplemental figures 3A3, 3B3, and 3C5, tuning strength would be far better to report than proportion significant.

We thank the reviewer for this suggestion, and now emphasize differences and similarities in tuning strength between conditions. The analyses in Figure 3 and S3 of the old manuscript are presented in Figure 4 of the revised version.

Minor Comments

6) The authors used a multisite recording electrode for their LGN recordings. An analysis of correlation in burst vs tonic activity across multiple simultaneously recorded neurons would strengthen the authors' message that pupil

size dynamics have a generalized relationship with LGN activity mode.

We thank the reviewer for this suggestion, and we are indeed actively exploring the topic of population activity. However, we believe that conducting a detailed analysis of population activity is beyond the scope of the current manuscript. Nevertheless, we have included an average burst cross-correlogram in this study to demonstrate that dLGN neurons exhibit a tendency to fire synchronously in bursts, displaying a rhythmicity of approximately ~ 5 Hz (Figure S2C). This observation provides initial evidence of coordinated activity within the dLGN population, which is relevant to our investigation of neural dynamics. However, we would also note that the diversity of timescales to which neurons couple most strongly (Figure S3C) suggests that activity modulations are not completely homogeneous across the population.

7) In multiple locations, the authors state "Bursts are driven by a hyperpolarization-activated Ca2+ current". This is not quite right, hyperpolarization does not activate the Ca2+ current; rather, hyperpolarization de-inactivates the T-type Ca2+ channel so that subsequent depolarization will activate the Ca2+ current.

We thank the reviewer for pointing out this error, and have corrected the text in the introduction.

8) line 41. "....cannot be fully characterized by a single assigning a single "level" of arousal to experimental epochs" Too many "single"s.

Thanks, this is corrected.

Reviewer 3

This manuscript aims to address an important question in the visual system - how is the dLGN neural firing dynamics modulated by the internal state of the animal? By decomposing the pupil size signals into a series of oscillatory components as represented by intrinsic mode functions (IMFs) and analyzing the relationship between individual IMF and the neural spikes, the authors revealed arousal-related modulation of dLGN neuron spiking activity across a broad range of timescales. Moreover, the spiking activity of dLGN units is modulated by the phase of multiple, simultaneously occurring pupil size oscillations, but cannot be explained solely by the pupil size. The neural bursts display stronger phase tuning as compared to the tonic spikes, and the two firing modes preferentially occur at opposing phases of pupil oscillations. Such pupil-associated modulation persists during the stationary period, suggesting a modulation effect independent from locomotion. The finer dissection of pupil-associated modulation across multiple timescales may provide important insights into the arousal-related modulation on sensory information encoding. However, I found it hard to evaluate the significance of the work. I am not convinced that the mathematical decomposition reflects meaningful biological processes. The neural basis of individual IMFs remains unknown. It is also unclear how stable and representative the IMFs are across different periods for the same recording units, across different units for the same mouse, or across different mice. I didn't see data showing whether results from analyzing individual IMFs are better than using the original pupil size signals without decomposition for revealing potential phase tuning. Moreover, the manuscript is highly technical and is overwhelming to review. I would urge the authors to rework the manuscript to improve the clarity, so that it is more digestible by the general readership of this journal.

Major comments

1) Before diving into individual intrinsic mode functions, did the authors analyze the association between dLGN spiking modes and the raw pupil size traces? Do tonic spikes tend to happen during pupil dilation and burst spikes occur more often during the contraction period when the pupil dilation and contraction is measured directly from the original pupil size dynamics?

We appreciate the suggestion and above we have analyzed dLGN firing rates modeled as a function of the temporal derivative of pupil diameter (Figure R2 on page 3). Our findings confirm that, as expected, the rate of change of pupil size captures some modulations in dLGN spiking - as does pupil size *per se* (Figure 1C). However, these analyses also reveal the shortcomings of these predictors, which further motivate the use of the pupil decomposition approach we have employed.

It is important to note that the terms "dilation" and "contraction" can be ill-defined when considering the original pupil size signal, as it is a complex and non-stationary signal. While taking the temporal derivative of the signal is one approach to extract these phases from the original signal, it may overly emphasize very rapid changes and introduce noise. Other authors have addressed this issue by first filtering the pupil size signal to focus on a certain frequency of oscillation [7], which provides more well-defined phases, but limits the conclusions to a specific timescale of pupil dynamics. In our study, we wanted to build on these analyses by considering more of the temporal scales present in the pupil signal. Our goal was to show that arousal-related modulation exhibits more complexity than what can be captured by a single variable, highlighting the importance of considering multiple temporal scales of modulation.

2) How stable and reliable are the characteristic frequencies of IMFs? Are the characteristic frequencies consistent between different pupil size segments, and between the periods of quiescence and locomotion? If the characteristic frequencies of IMFs indeed have biological correspondence, such as certain neuromodulatory mechanisms, shall we expect that the neuromodulation is acting on a relative stable timescale and therefore the IMFs are also stable across time and across mice?

We thank the reviewer for these interesting questions. It is important to note that the decomposition does not provide components that can be matched across different decompositions. When computing the marginal power spectrum from the components, we observe a diversity of shapes across recordings, as reflected by the broad distribution of the highest power components in Figure 1F. That said, the average dynamics across mice are similar between quiescence and locomotion (Figure R6 on page 10). It is crucial to clarify that these components do not necessarily have biological correspondence; rather, they serve as a tool to separate the temporal scales present in the pupil signal (see Discussion). In both the introduction and discussion of the new version of the manuscript, we emphasize that internal and behavioral states undergo changes over multiple temporal scales. Consequently, our analysis aims to capture the concurrent neural modulations that reflect this temporal complexity.

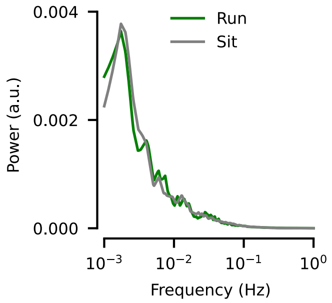

Figure R6 Mean power spectral density of the pupil size signal – derived via the Hilbert-Huang Transform [4], as in our manuscript – for periods of quiescence and locomotion

3) The results from different PS segments/units were simply pooled together at multiple places, eg. Figure 1F, Figure 2D-F. But we do not know the results grouped by the recording unit or animal identity. Therefore, it's hard to assess how consistent the results are across samples.

Thank you for raising these questions. We now split the data from Figure 2 across recording sessions and present the results in Figure S3C. Overall, we argue below that the phase preferences are quite consistent across the whole data set (see response to point 4)), but that the timescales to which units most strongly couple is diverse across neurons (see response to point 5)).

We would also like to add that we have, during the course of the revisions, substantially improved some details of the data pre-processing. We now only include one long, high-quality segment of data from each recording, reducing the overall number of observations but increasing the quality of their contribution. In addition, this enables a more accurate assessment of longer temporal scales of modulation and mitigates statistical issues arising from hierarchical nesting of observations.

4) What's the distribution of the phase tuning preferences across different units in the same animal, under the same IMFs? What's the distribution of tuning strengths? Are these distributions consistent across mice? What about the phase tuning for bursts and tonic spikes across different IMFs for the same unit? How to interpret these results?

We would like to highlight that the preferred phase (Figure 2C), and the 180° phase relationship between tonic spiking and bursting (Figure 2D), is highly consistent across neuron-component pairs. Therefore we would argue that it must also be largely consistent for 1) the several neurons tuned to the same component, 2) the several components to which a single neuron is tuned, and 3) across neurons and components from different animals. For a discussion on the shift of preferred phase across timescales, see our reply to Reviewer 1, point 5).

We discuss coupling strengths below.

5) For different units recorded in the same animal, what's the distribution of the characteristic frequencies of the IMFs showing the strongest phase tuning? If different units of the same animal show strong phase tuning at different characteristic frequencies, is the choice of the frequency related to their visual response properties? Please discuss the potential mechanisms.

We have added the requested distributions for each recording session in Figure S3C2 and S3C3. In contrast to the consistency of phase preferences across neurons, individual neurons have diverse preferred timescales. We hope that illustrating these distributions will allow for a better understanding of the range of modulation types observed.

These diversity in coupling strengths and preferred timescales may arise due to various factors, such as the visual response properties of the neurons, their anatomical location, behavioral involvement, or simply the stochastic distribution of modulatory inputs. We have incorporated these points into the discussion section.

The question regarding the relationship between preferred stimulus temporal frequency and preferred frequency of modulation by internal state is very interesting, especially given recent results by the groups of Bonin and Anderman, showing that running-related response modulations in dLGN can depend on the visual response preferences. Our lab is actively investigating these questions. However, in the present set of experiments, we were not able to sample the relevant stimulus dimensions, in particular spatial and temporal frequency tuning.

6) The correlation (r2) in Figure 3 A1-2, B1-2, C1-2 is tiny. Given the small r2, the claim that one variable can 'somewhat' predict the other variable is misleading. Although the p value is small, the small p is due to a large number of data points and the p value only reflects the confidence of the correlation coefficient. For these reasons, I am not convinced that there was a significant correlation between the variables.

We completely agree, and indeed we intended with these panels to demonstrate a lack of correlation in each of these cases. However, we also felt that it was good practice to report the results of the statistical tests. To address the issue raised by the reviewer above and the difficulty in interpretation, we have replaced these analyses with new ones, which we now present in Figure 4 and S4. While this change does not affect our main conclusions, we believe that it strongly improves the clarity of our main message.

7) In the dataset for measuring the correlation between firing mode and pupil size dynamics, 3 mice were displayed with a static gray screen while 16 mice were stimulated with sparse noises. It is unclear whether the analysis of firing mode was constrained to the temporal segment when no luminance level change occurred in the receptive

field of the units. If not, how to make sure that the firing mode was not directly influenced by the visual stimulation?

We did not exclude periods of time where a stimulus was presented in the receptive field of the neurons. Our reasoning behind this decision was twofold: 1) this happens relatively rarely, and 2) because the sparse noise stimulus was globally isoluminant, any local luminance change in the receptive field should be statistically independent of pupil size changes. Therefore, we believe that we are still able to measure phase coupling during the presentation of this stimulus. However, we acknowledge potential caveats. As a result, we have since the original submission made an effort to include a more balanced data set with more recordings performed in front of a static gray screen (Gray screen N = 5 sessions in 3 mice, Sparse noise N = 10 sessions in 8 mice). This allowed us to separate the phase coupling results from these two conditions (Figure R7 on page 12), revealing that patterns of phase tuning in the two conditions are nearly identical.

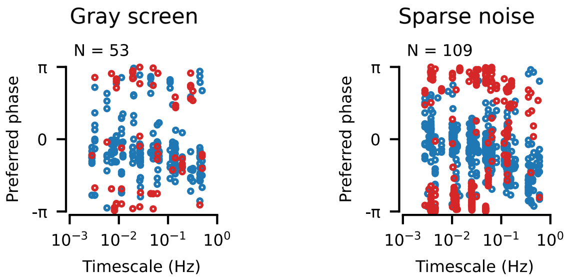

Figure R7 A comparison of phase coupling measured using gray screen and sparse noise visual stimuli.

8) The relationship between the locomotion-dependent modulation and pupil size-dependent modulation is still unclear. If the EMD decomposition is applied to the locomotion signals and then similar analyses are applied to the locomotion IMFs and firing dynamics, could a similar phase-firing mode relationship be observed?

We agree with the reviewer that our previous version of the manuscript left the relationship between locomotion-dependent modulation and pupil-related modulations somewhat unclear. We have now fundamentally revised the portion of text dealing and figures with the contribution of locomotion (Figure 4 and S4). In brief: Given that parts of the pupil dynamics are correlated with running speed it is indeed expected that a similar relationship would be observed if we were to apply our phase coupling analysis with running speed rather than pupil dynamics. However, our new analyses show that a component of pupil dynamics is not more likely to be associated with significant tuning simply because it is correlated with running speed. In general, using pupil dynamics rather than run speed for the decomposition has several advantages. Firstly, the pupil dynamics can capture modulations even when run speed is consistently zero. Secondly, from a technical standpoint, applying the decomposition to a signal such as run speed poses challenges due to its burst-like nature, i.e. with many periods lacking any dynamics whatsoever.

Minor comments

I found at multiple places that the writing was not straightforward and the underlying logic was obscure. A number of these places are listed as follows.

We completely agree with the reviewer that the text contained too many technical details and that the writing was not straightforward. We have therefore completely revised the manuscript to improve readability and focus on biological interpretations rather than methods.

1) Line 95-97: 'Lastly, we found that these IMFs tended to have weak linear, higher-order, and phase relationships (Figure S1F-H), indicating that they captured largely independent cycles of pupil dilation and contraction across a broad range of timescales.' I am confused by 'have weak linear, higher-order, and phase relationships'. I am not convinced that the different phases of individual IMFs can be interpreted as 'pupil dilation and contraction', as some of the IMFs look really different from the original pupil size signals, for example, the IMF at 0.012 Hz in

Figure 1C.

We agree with this comment and apologize for the confusion. We have now developed a single, more straightforward analysis. The terms "linear", "high-order", and "phase" were originally used to describe the results of the correlation, ICA, and spectral coherence analyses between components of pupil dynamics shown in the previous version of Figure S1. These analyses aimed to demonstrate that the components obtained from a decomposition are unlikely to have strong phase relationships to each other, which could lead to spurious coupling of neural activity to multiple components.

Our new analysis addresses this question much more directly, by checking if, and for which combination of phases exactly, two components have the same phase (Figure S1D). This allows us to identify specific time periods when two components are in the coupled phase combination. In Figure S3B, we now show that neurons are still coupled to multiple components, even after excluding time periods where two components might exhibit phase coupling. Additionally, we have now clarified that phases of the components do not necessarily correspond to actual dilation and contraction of the pupil, but represent an underlying aspect of the dynamics.

2) Line 131-133: 'Together, these results indicate that pupil size oscillations index multiple timescales of firing mode modulation, which coexist across the population of dLGN units, as well as within the spiking activity of single units.' I am confused by 'which coexist across the population of dLGN units, as well as within the spiking activity of single units.'

Please see reply to point 3 below.

3) Line 160-161: 'We conclude that dLGN units alternate between a tendency to fire in bursts during pupil contraction and tonic spikes during pupil dilation over multiple timescales.' Does 'over multiple timescales' apply only to the population result, or also to individual units? I'd like to see paired results of the preferred phases of burst firing vs. tonic firing under the same IMF and for the same unit. The IMFs can be the ones with the strongest power and the ones with the strongest phase tuning, respectively.

We apologize for the confusing caused by our previous wording. We have now revised the text to be more precise about what is meant by "timescales... coexist" and "over multiple time scales". Firstly, we find that different units can be preferentially coupled to different temporal scales (Figure S3C), suggesting that different neurons may be more responsive to certain timescales compared to others. Secondly, we find that individual neurons can exhibit significant tuning to more than one temporal scale (Figure 2F), showing that a single neuron may be sensitive to more than one timescale of modulatory influences.

Regarding the requested figure showing the phase of coupling of burst versus tonic spikes to each component, it was originally already included in Figure 2 and can now be found in panel 2D. To prevent that it will be missed in the future, we have also made efforts to provide a clearer description of this figure panel in the caption and main text.

4) Line 174: 'its overall influence was small (bursts: slope = 1.7x10-3; tonic spikes: slope = 6.8x10-4)'. If 'the influence was small' was based on the slope, the slope value needs to be normalized because the units for the x and y axis are very different.

We agree and have removed these analyses.

5) Line 175-177: 'Such a small, positive relationship might be at least partially explained by the fact that, in general, phase estimation for low power components will be less reliable, and therefore phase tuning measured for these components will be lower.' The logic underlying this statement is unclear. Given that the correlation is very small, it's not convincing that phase estimation for low power components will be less reliable. On the other hand,

if we can take this conclusion, why didn't the authors simply choose the IMFs with the highest power to display the phase tuning results in Figure 2 since the IMFs with highest power were the most reliable?

We acknowledge that the correlation was small and hard to interpret and appreciate the feedback. As a result of this and similar comments by the other reviewers, we have decided to remove this analysis entirely from the manuscript. However, we continue to analyze all of the components because we do not have knowledge about the precise power at which the phase becomes unreliable. If the phase is sufficiently unreliable, it will simply result in non significant coupling, which we do not consider for some of the further analyses.

6) Line 201-202: 'Having observed that a number of IMFs were significantly correlated with running speed (37%, 100/270 IMFs; Figure S3B1, black dots)'. For these cases, what's the correlation between running speed and the raw PS? Does the running speed correlate more with individual IMFs compared to the raw PS?

We have included the requested analysis below (Figure R8 on page 14), showing that most components correlate with locomotion to a smaller degree than the original pupil size signal from which they derive.

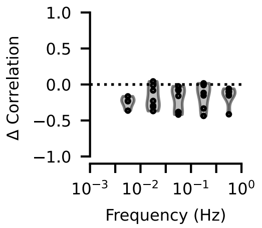

Figure R8 A comparison of the correlation of pupil dynamics components that are significantly correlated to run speed (separated by timescale), with the correlation of the original pupil size signal and run speed.

7) Line 203-206: 'We found that correlation with running speed was somewhat predictive of IMF phase tuning strength for bursts (F2 = 7.85, p = 4.06 10-4; Figure 3B1), and tonic spikes (F2 = 11.61, p = 9.51 10-6; Figure 3B2), though the amount of variance explained was minimal in both cases (bursts: r2 = 9.30×10^{-3}; tonic spikes: r2 = 8.60×10^{-3})'. Given the extremely small r2 values, the statement that 'the correlation with running speed was 'somewhat' predictive of IMF phase tuning strength for bursts' is misleading.

We agree, and have removed this analysis from the manuscript as it is too difficult to interpret. We now emphasize the finding that although these correlated components exist, they are not more likely to drive tuning (Figure S4). Additionally, we highlight that even during quiescence, when there is no locomotion speed signal, we still find coupling to the components (Figure 4).

8) Line 278-279: 'our results suggest that dLGN stimulus responses occur on top of arousal-induced modulations in spiking mode, which could have profound consequences for the type of information that is passed on to the cortex.' It is unclear what 'the type of information' refers to here.

We thank the reviewer for pointing this out, we have re-worked our description of stimulus encoding by bursts to be more precise about what is meant (see Discussion). We would also highlight our own substantially expanded analysis of stimulus encoding differences across phases of pupil dynamics (Figure 5).

9) Line 281-282: 'Our results establish that spiking mode in dLGN, the primary visual thalamic nucleus, is predicted by pupil size dynamics occurring over multiple timescales'. This statement is also confusing. Could the spiking mode of an individual unit be predicted by several IMFs of the same pupil size segment? Or is this statement

referring to the population result?

We apologize for the vague wording. This statement is no longer present in the text, in favour of less ambiguous descriptions.

References

[1] de Gee JW, Tsetsos K, Schwabe L, Urai AE, McCormick D, et al. (2020) Pupil-linked phasic arousal predicts a reduction of choice bias across species and decision domains. Elife 9: e54014.

[2] Erisken S, Vaiceliunaite A, Jurjut O, Fiorini M, Katzner S, et al. (2014) Effects of Locomotion Extend throughout the Mouse Early Visual System. Current Biology 24: 2899–2907.

[3] Hess EH, Polt JM (1964) Pupil size in relation to mental activity during simple problem-solving. Science 143: 1190–1192.

[4] Huang NE, Shen Z, Long SR, Wu MC, Shih HH, et al. (1998) The empirical mode decomposition and the Hilbert spectrum for nonlinear and non-stationary time series analysis. Proceedings of the Royal Society of London Series A: mathematical, physical and engineering sciences 454: 903–995.

[5] McCormick DA, Nestvogel DB, He BJ (2020) Neuromodulation of brain state and behavior. Annual review of neuroscience 43: 391–415.

[6] Niell CM, Stryker MP (2010) Modulation of visual responses by behavioral state in mouse visual cortex. Neuron 65: 472–479.

[7] Reimer J, Froudarakis E, Cadwell CR, Yatsenko D, Denfield GH, et al. (2014) Pupil fluctuations track fast switching of cortical states during quiet wakefulness. Neuron 84: 355–362.

[8] Reimer J, McGinley MJ, Liu Y, Rodenkirch C, Wang Q, et al. (2016) Pupil fluctuations track rapid changes in adrenergic and cholinergic activity in cortex. Nature Communications 7: 1–7.

[9] Schriver BJ, Bagdasarov S, Wang Q (2018) Pupil-linked arousal modulates behavior in rats performing a whisker deflection direction discrimination task. Journal of Neurophysiology 120: 1655–1670.
