# Supplementary material for: Spiking activity in the visual thalamus is coupled to pupil dynamics across temporal scales
Source: PLoS Biol. 2024 May 14;22(5):e3002614. doi: 10.1371/journal.pbio.3002614 (PMC11093384; doi:10.1371/journal.pbio.3002614)
Supplement: Attachment — Submitted filename: Crombie_et_al_rebuttal.pdf [file pbio.3002614.s009.pdf]

Dear Dr. Smith, dear editorial team, dear reviewers,

We thank you very much for the continued interest in our work and the opportunity to revise our manuscript entitled "Spiking activity in the visual thalamus is coupled to pupil dynamics across temporal scales". We found the reviewers' comments very insightful and constructive, and feel that they have greatly improved the quality of the manuscript.

Here is an overview of the major changes to the manuscript:

1) As suggested by both reviewers, we now report more raw data. For instance, we have replaced the modeled firing rate in Fig. 1C with spike counts, and further added the requested analyses of burst rates (Fig. S3D). We have also extended the example trace showing phase coupling of burst and tonic spikes in Fig. 2B, where we enhanced the graphics to make sure it is possible to visually distinguish times with high tonic spiking.

2) Prompted by comments from Reviewer 2, we have rephrased the manuscript throughout to make clear that state-related modulations of firing rates in dLGN could be elicited by multiple mechanisms, from neuromodulatory inputs to re-afferent sensory input due to changes of retinal illumination associated with changes in pupil size. Similarly, we have clarified that trial-by-trial variability of dLGN activity in response to the repeated movie presentations can arise from a variety of mechanisms associated with changes in internal state. We think that these changes greatly enhance the clarity of our main message: that dLGN spiking activity is modulated at multiple temporal scales, from several minutes to seconds.

3) In response to questions by Reviewer 2, we have removed figure panels showing the number of timescales to which tonic and burst spikes are coupled, as we concluded that these numbers cannot be directly compared. This is because the statistical test to determine significance will depend on effect size (i.e. tuning strength), which we show differs between the spike types.

We have also implemented the remaining suggestions, which are detailed in the point-by-point response to the comments below. In particular, we have taken great care to perform the additional analyses suggested by Reviewer 1 to demonstrate the specificity and reliability of the EMD. We hope this additional evidence convinces the reviewer that the EMD analyses enables useful insight into multi-scale modulation of dLGN spiking by internal state. We have also made sure to further simplify the language and illustrations where applicable, and have enhanced the consistency of the statistical reporting. All changes to the text in the manuscript are highlighted in blue.

Reviewer #3: I would like to thank the authors for thoughtfully considering my questions and providing informative answers. Both the quality of the data analysis and the writing of the revised manuscript has been greatly improved. However, it still took a significant amount of efforts for me to read and digest the manuscript, due to the complexity of the data analyses, inadequate explanations of the methods, and typos in the text. Moreover, some of my previous questions were not adequately addressed or given clear explanations.

We thank the reviewer for the positive evaluation of our previous revision. We hope that we can address the remaining issues with this revision and the replies below.

Major comments:

1. I am convinced now that the multiple timescale analysis of pupil dynamics provides valuable new insights into the modulation of dLGN visual processing by behavioral state, and there is something interesting and robust in the coupling of the firing mode with the phase of pupil dynamics. However, I am still not sure about the reliability of the phase coupling for individual neurons or the decomposition of pupil dynamics by empirical mode decomposition (EMD). For example, if the segment data from each recording is separated into two halves, and the CPD and coupling analysis is applied to each half, shall we expect similar results regarding the timescale that gives the strongest coupling and the preferred coupling phase for burst vs. tonic firing at each timescale?

We thank the reviewer for the suggestion. To assess the robustness of the coupling we split spiking activity from each neuron into two halves to compare phase coupling to CPDs (which are still extracted from the full recording segment). We assessed phase coupling separately in each half and observed overall similar results when compared to the analysis using all spikes. Specifically, we found a substantial proportion of neurons for which the strongest

coupling CPD was the same or directly adjacent in frequency such that, across the two halves of the recording, ≥80% of neurons either retained the same coupling timescale or switched to the next most similar timescale (Figure R1A; tonic spiking: 80.0%; bursting: 88.6%). Finally, coupling phases were also robust: for neuron-CPD pairs with significant tuning in both halves, the coupling phases were similar across halves (Figure R1B); mean phase difference across halves for tonic spiking: -0.01; bursting: -0.12).

To test whether this small variability in coupling is inherent or comes from slow changes across the recording, we performed a second analysis where the data was split into two halves, but this time in an interleaved manner, i.e. assigning spiking activity from adjacent cycles of the CPD to different groups. We obtained similar results with an interleaved split (Figure R1C-D; mean phase difference across halves for tonic spiking: 0.13 bursting: -0.10). The similar distributions of timescale preference and phase shifts for these "interleaved splits" compared to the case when spiking was split between the first and second half of the recording shows that the small amount of variability in the former is likely not due to slow changes over time.

More generally, the observed shifts in strongest CPD by 0 or 1 timescales should be interpreted in light of our observation that, for neurons with coupling to multiple timescales, the two with the strongest coupling tend to be adjacent (Figure R1E). Overall, these results show that our phase coupling framework robustly assigns neuronal coupling to a narrow band of temporal scales.

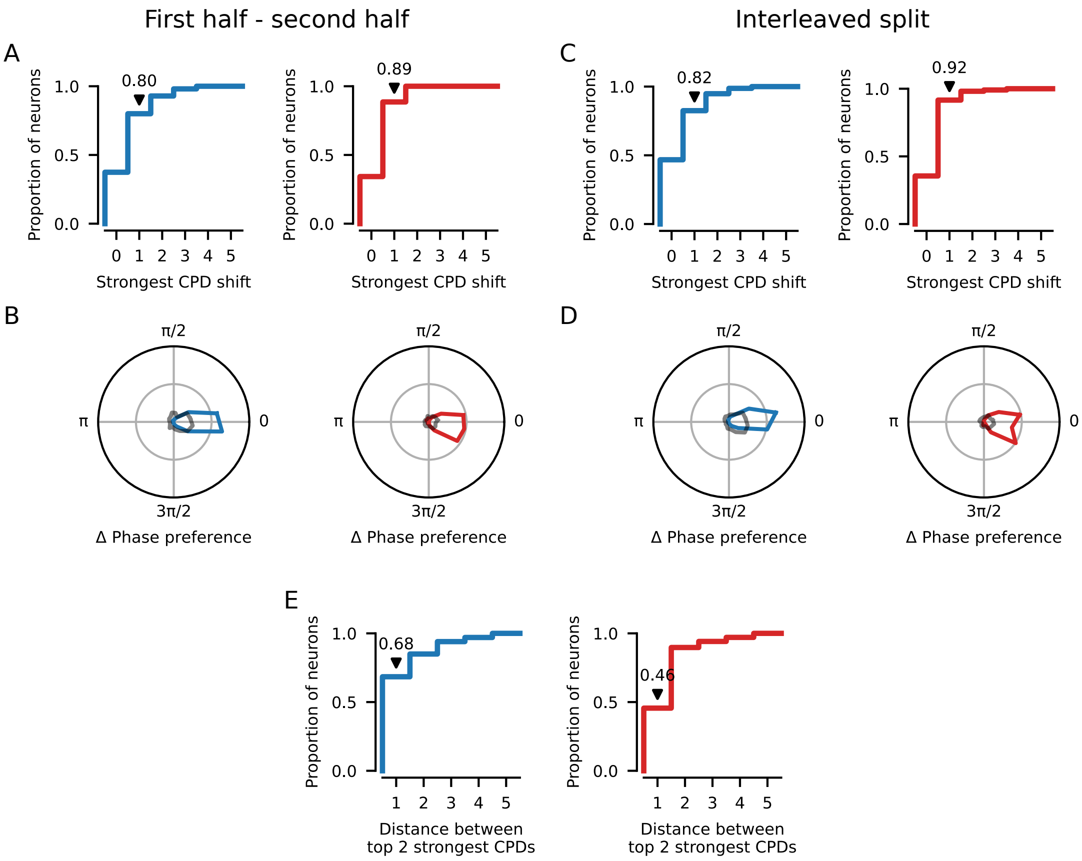

Figure R1 **(A)** Cumulative distribution of the shift in preferred timescale (stongest CPD) for each neuron in the first half of the recording versus the second half (*blue*: tonic spiking, *red*: bursting). **(B)** Distribution of the difference in coupling phase for each neuron-CPD pair in the first half of the recording vs. the second half (*colored*: neuron-CPD pairs with significant coupling in both halves, *gray*: neuron-CPD pairs with non-significant coupling in either of the two halves, *grid lines* indicate proportion of 0.25). **(C, D)** Same as (A, B) but splitting spiking activity from adjacent cycles of the CPD to different groups ("interleaved split"). **(E)** The distance between the two CPDs with the strongest coupling, for all neurons with coupling to more than one CPD for tonic spikes (*left*) and bursts (*right*). For this analysis, coupling was assessed after removal of periods of synchrony between CPDs (as in Figure S3B).

... Similarly, if the segment of data from the same unit is separated according to behavioral states, will similar coupling results be obtained across behavioral states?

Yes. We find that regardless of behavioral state, coupling of tonic spikes to CPDs have similar timescale preferences (Figure R2A), and a robust coupling phase (Figure R2B, left). Bursting, however, showed more variable phase preferences across behavioral states (although the difference between states consistently remained less than 90°; Figure R2B, right). This variability aligns with the broader distribution of burst-tonic phase differences during running seen in Figure 4B3.

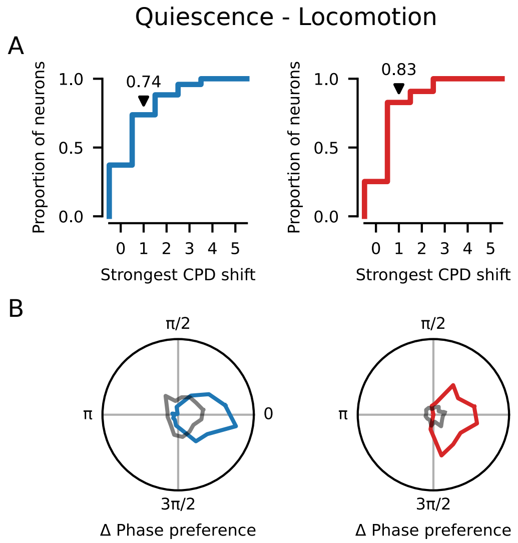

Figure R2 (A) Cumulative distribution of the change in preferred timescale ("strongest CPD") for each neuron during quiescence versus locomotion (*blue*: tonic spiking, *red*: bursting). **(B)** Distribution of the difference in coupling phase for each neuron-CPD pair during quiescence versus locomotion (*colored*: neuron-CPD pairs with significant coupling in both states, *gray*: neuron-CPD pairs with non-significant coupling in either of the two states, *grid lines* indicate proportion of 0.25).

2. The authors now showed that the timescales to which units are most strongly coupled are diverse across mice and across sessions (Figure. S3C3 and S3C4). Although the diversity of dLGN neurons may be the reason underlying this result, another possibility for the result is a lack of reliability or specificity of the EMD analysis.

We believe that such differences between recordings are expected due to the inherent diversity of the pupil signal power spectral density function across sessions, irrespective of the method used to measure it. Evidence that diversity in coupling preferences can arise from biological differences between sessions/ mice comes, for instance, from analyses in which we found a strong dependence of the mean preferred coupling timescale of tonic spiking on the frequency of behavioral state switches in the session (Figure R3A; Pearson's r = 0.71, $p = 4.6 \times 10^{-4}$, N = 14 sessions). This is not the case for the timescale preferences of bursting (Pearson's r = -0.13, p = 0.66, N = 14). Regardless, this result supports the interpretation that the diversity in coupling preferences is not an artifact of an imprecise decomposition, but can reflect measurable factors. We believe this is an important point, and have therefore also included the reviewer figure in the manuscript (as Figure S3C).

In general, we appreciate the reviewer's concern regarding the EMD method as it is not canon, but we would like to note that it is a well-established signal decomposition method; the keystone publication [3] has been cited over 28,000 times across scientific disciplines. The method is considered reliable in the sense that it gives similar results as more traditional methods where applicable. For example, the marginal power spectra obtained via HHT (the combination of EMD and Hilbert spectral analysis applied in our manuscript) closely resemble those obtained using the Fourier transform (Figure R3B). In addition, if there is a strong oscillation present in the signal, EMD extracts a component at this frequency (see, e.g. [5], for an application to hippocampal theta). While yielding similar results in some aspects, HHT outperforms Fourier transform or wavelets for signals with unknown or variable underlying phase progressions, as it is capable of capturing cycle-to-cycle differences in the phase progression of an extracted component [8]. This is the case for the pupil signal (see [7] for an application of EMD to the pupil size signal in humans), which motivated us to explore the use of EMD in our manuscript.

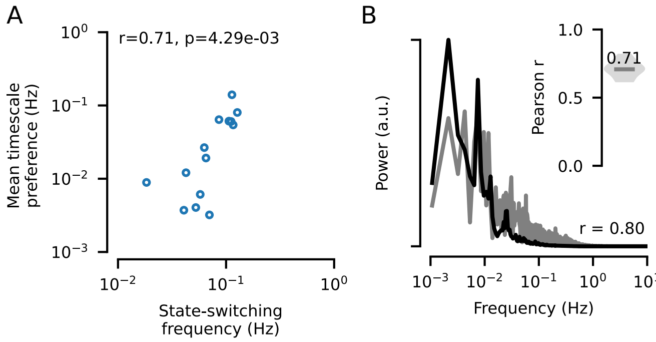

Figure R3 **(A)** The mean timescale preference across neurons in each recording session plotted against the frequency of behavioral state switches frequency (1 / mean duration of run and sit periods) in each recording (Pearson's r = 0.71, p = 4.6×10^{-4}, N = 14 sessions). **(B)** The marginal power spectral density for the example pupil size trace in Figure 1 as measured using the HHT (*black*) and the FFT (*grey*). The Pearson correlation is high (r = 0.80) indicating a good correspondence. *Inset*: distribution of FFT-HHT PSD correlations for all recordings (median r = 0.71, N = 15 sessions).

3. In the answer to my original question 1, the authors refer to Figure R2. But Figure R2 does not look to be relevant. I suspect the authors actually referred to Figure R3. The pupil size gradient should have both negative and positive values. But it's unclear whether the normalized pupil gradient in Figure R3 was based on the absolute gradient, or whether 0 and 1 corresponded to the most negative and most positive values. Moreover, I asked about the relationship between the firing mode and the change of pupil sizes. Figure R3 showed the dLGN spike rate (spike count in 250 ms bin?) but not the firing mode.

We apologize for the incorrect figure reference in our previous reply and that we did not address the reviewer's question in a satisfactory way. Below, we provide the figures showing the relationship of each spike type with the pupil size gradient along with the suggested improvements (Figure R4). In the previous reply, a gradient value of 0 was the most negative, and 1 the most positive. As suggested, we now express pupil size gradients normalized by the maximum absolute value from that session, but retaining the sign. We emphasize that neurons show diverse profiles of modulation in their tonic spiking (Figure R4A; 80.1% of neurons had tonic spike rates significantly modulated by the pupil size gradient, ANOVA across 20 gradient bins $p \leq 0.05$). As requested, we now also include the same analysis for burst rates, and found that a relatively small proportion of neurons (37.2%) had burst spiking modulated by the pupil size gradient. Of those, many had the highest burst rates at a gradient of zero (Figure R4B). This is expected from the results reported in Fig. S3D and Fig. S3D of the manuscript showing that dLGN bursting is partly related to pupil size *per se*.

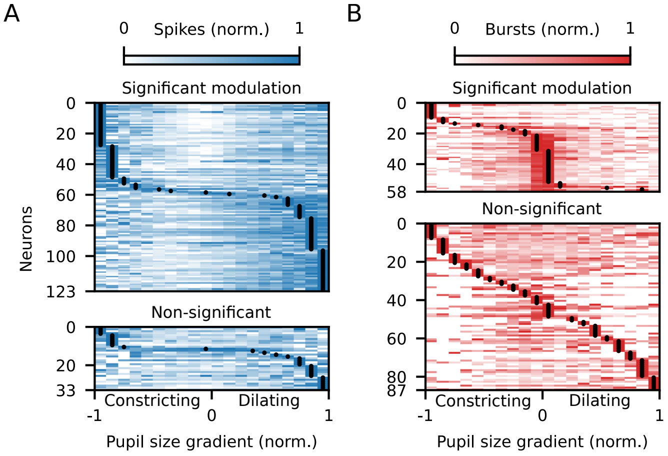

Figure R4 **(A)** Tonic spike rates as a function of the pupil size gradient for all recorded dLGN neurons (*top*: neurons with significant modulation of firing rates across gradient bins (ANOVA $p \leq 0.05$), *bottom*: neurons without significant modulation). **(B)** Same as (A) but for burst rates.

4. In Figure R7, no stats were performed to compare the distributions of the preferred phase under gray screen vs. sparse noise stimulation conditions. The preferred phase of the bursting firing under the gray screen condition

actually looks to have a larger fraction concentrated at 0 deg phase, different from that under the sparse noise stimulation condition.

We apologize for the lack of statistical tests in our previous response, below we provide the distributions and statistics for tonic spike and burst coupling phases. For tonic spiking, we observe that the mean coupling phase differed between the two experimental conditions (Figure R5A; mean phase gray screen = -0.71, sparse noise = -0.46, Watson-Williams: $F_{1,680} = 8.51$, $p = 3.66 \times 10^{-03}$). One reason for this difference could be the greater number of long-duration recordings in the sparse noise condition (# of recordings > 1000 s gray screen = 2/5, sparse noise = 9/10), allowing the measurement of coupling to longer timescales where the coupling phase tends to be shifted more towards 0 (see Fig. 2C). For burst spiking, there was no significant difference in the mean coupling phase (Figure R5B; mean phase gray screen = -2.68, sparse noise = -3.00, Watson-Williams: $F_{1,318} = 0.52$, $p = 0.47$), and the difference in the coupling phases of bursts and tonic spikes was identical between gray screen and sparse noise conditions (Figure R5C; tonic - burst coupling phase mean difference gray screen = 2.63, sparse noise = 2.83, Watson-Williams: $F_{1,282} = 0.95$, $p = 0.33$). Finally, coupling strengths did not significantly differ between conditions for tonic spiking (gray screen median = 0.0034 vs. sparse noise median = 0.0052, Mann-Whitney-U test: $U = 8.5 \times 10^4$, $p = 0.13$). Sparse noise coupling strength were higher for bursting (gray screen median = 0.0068 vs. sparse noise median = 0.0118, Mann-Whitney-U test: $U = 4.7 \times 10^4$, $p = 0.02$). Again, this is likely due to the inclusion of longer recordings (burst coupling strengths are higher for slower timescales, see Figure 2E) as there is no significant difference for only the faster half of the CPD timescale range ($U = 3.0 \times 10^4$, $p = 0.57$). We feel that these results support our pooling of data from sparse noise and gray screen conditions.

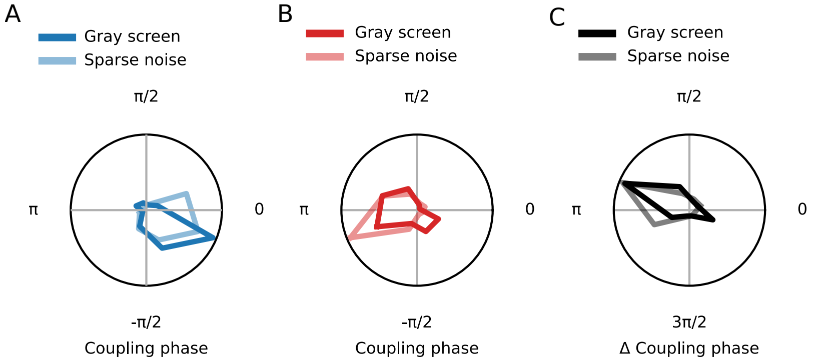

Figure R5 **(A)** Distribution of tonic spike coupling phases for all neuron-CPD pairs recorded under gray screen stimulation (*bold line*) and sparse noise stimulation (*faded line*; mean phase gray screen = -0.71 vs. sparse noise = -0.46, Watson-Williams: $F_{1,680} = 8.51$, $p = 3.66 \times 10^{-03}$). **(B)** Same as (A) but for burst coupling phases (mean phase gray screen = -2.68 vs. sparse noise = -3.00, Watson-Williams: $F_{1,318} = 0.52$, $p = 0.47$). **(C)** Distribution of tonic spike - burst coupling phase differences for all neuron-CPD pairs recorded under gray screen stimulation or sparse noise stimulation (tonic - burst coupling phase mean difference gray screen = 2.63 vs. sparse noise = 2.83, Watson-Williams: $F_{1,282} = 0.95$, $p = 0.33$).

Other minor comments:

1. Line 74-75: 'However, we also noticed that similar ranges in pupil size fluctuations occurred within both active and quiescent behavioral states (Figure S1B3).' Please provide a movie clip of the pupil recording and the trace of simultaneously recorded locomotion speed for the period when pupil reached the minimal area in the displayed range in the figure and when the animal was running.

We believe the reviewer is noting that it is unexpected to have a small pupil during locomotion. After inspection, we noticed that all instances of a small pupil (pupil area in the lowest quartile of the observed range) during locomotion occur when the animal is engaged in slow (1-5 cm/s), but sustained (>2 s duration) locomotion. We have provided an example movie clip, with the corresponding traces in Figure R6. We use a 1 cm/s speed threshold for detecting periods of locomotion, in line with previous publications [6, 9, 11]. Thus, we do not feel it is appropriate to change our criteria for detecting bouts of locomotion. However, following the above, we believe it is appropriate to always use the more general term "locomotion" instead of "running", and now do so in the manuscript.

However, with regards to the specific figure panel referred to by the reviewer, we found that this panel was inappropriate to underscore the point we were trying to make. Rather than comparing the pupil size *range* between behavioral states, we find it more appropriate to compare that the pupil size *dynamics*, which we now quantify

using power spectral density in Figure S1B$_5$.

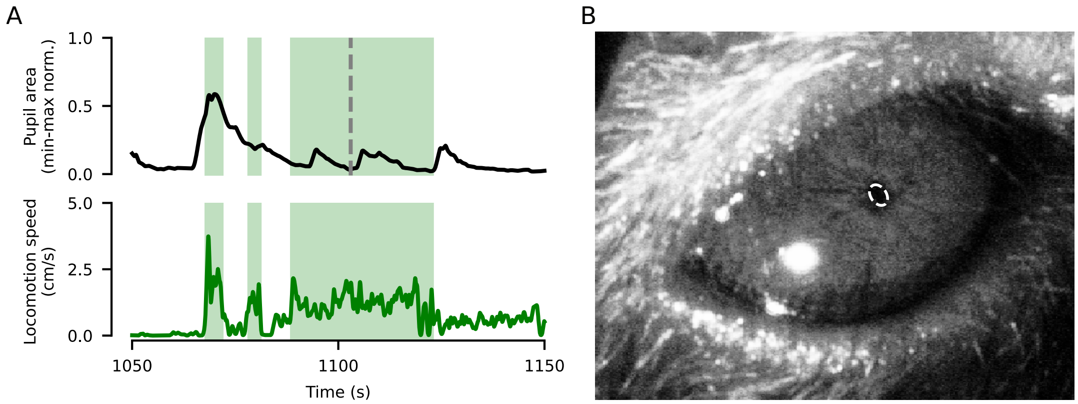

Figure R6 (A) *Top*: Pupil area (min - max normalized; *dashed line*: example video frame). *Bottom*: Locomotion speed (*green shaded regions*: detected locomotion bouts). **(B)** Example video frame indicated in (A) (*dashed white ellipse*: ellipse fit to pupil).

2. Possible typos: Line 161: Figure S1E was referred to in the text. But there is no Figure S1E.

Line 168 -169: Figure S3C2 and S3C3 should be S3C3 and S3C4.

Line 171: Figure S2C2 and S2C3 should be S3C3 and S3C4.

Our apologies, all of the above have been adjusted.

New Reviewer #2 (who assessed the responses to Reviewers 1 and 2): The study investigates in the mouse the interplay between neural activity in the visual thalamus and pupil dynamic. The pupil controls retinal illumination in response to changing light levels but also reflects behavioral variables such as arousal and locomotor activity, which correlate with neuromodulatory processes critical for sensory processing. The paper argues that spontaneous visual thalamic activity shows multiscale temporal coupling to pupil dynamic, occurring preferentially at specific temporal phases across various frequencies. Tonic spikes and bursts show opposite phase preferences, with activity located preferentially at the peaks and troughs of the pupil the size dynamic (the effects are more pronounced for bursts than tonic spikes). This coupling and phase tuning is observed for diverse time scales in constant illumination, darkness, and upon visual stimulation. The temporal relationship is robust and not explained by behavioral variables such as locomotor activity and saccadic eye movements and is proposed as an organizing principle of visual thalamic activity.

Overall, this interesting, high-quality study adds to our understanding of how visual neurons respond during behavior. While the investigation is focused on ongoing activity, the paper does examine responses to naturalistic movies, exploring the interplay between ongoing and visually-evoked activity.

Although the analytical methods are involved, the paper is well written, and the results clearly presented. The authors have gone to great lengths to address the reviewers' concerns by adding new analyses and data and making comprehensive textual changes to the manuscript. The authors' response to the reviewers' is overall satisfying.

We thank the reviewer for their interest in our study.

However, issues of analysis and interpretation remain from the original submission and new issues were added in course of the new revision.

Main comments:

1. The manuscript has repeated mentions of 'nested' temporal dynamics. However, readers may not be familiar with this concept (I am not). The term is used in reference to arousal in the Introduction (line 53) but is not defined. There is a sentence in results (lines 153-154) referring to nested modulations, but again, it is unclear what is meant. If the word is to be used, the authors should clearly explain it in the Introduction and explain what aspects of the data support the hypothesis (or provide evidence against it).

We appreciate that the term can be ambiguous, and have therefore removed it from the manuscript in favor of more concrete descriptions.

2. Multiple statements are also made about neurons being tuned to fewer or more time scales in different experimental conditions. For instance, Fig. 4 shows eye movements and quiescence don't influence coupling, but locomotion seem to have a significant effect. How should these differences be interpreted within the proposed framework of nested modulations?

We thank the reviewer for this comment, but due to considerations addressed below (point 3), we no longer feel it is appropriate to interpret these results, and have limited the extent to which we do so in the manuscript.

3. At a practical level, how sensitive is the analysis to the number of events detected? The bursts are very sparse, which could limit the potential for observing coupling at fast temporal scales. Are the differences in coupling and number of coupled frequencies reported for bursts and spikes (e.g., Fig. 1E-F) driven by the relative rarity of bursts? Would the analyses of the coupling of tonic spikes yield the same results if the number of spikes and bursts were matched? If the blue and red curves cannot be compared because of limitations in the data set, this should be stated explicitly.

This was one of our main concerns when developing our phase coupling analysis. The conventional statistic used to assess the concentration (variance^{-1}) of a circular variable, the 'mean resultant length' (MRL), is prone to bias, tending towards higher values when N is small. To avoid this bias, we employed the squared MRL as a measure of coupling strength, a choice that allows for straightforward bias correction, as described by [4]. Thus, our measure of coupling strength (Figure 2E) is not influenced by the number of events detected.

However, a small number of observations has the unavoidable consequence of reduced statistical power. A smaller number of bursts could thus result in a smaller number of tests reaching significance level (former Figure 2F), and the number of CPDs to which burst spiking is coupled is indeed correlated to the number of bursts observed. While one could opt to sub-sample tonic spikes to match the number of bursts and assess phase coupling using these sub-samples for comparison, it is crucial to acknowledge that statistical power is also influenced by effect size (i.e. coupling strength). Given that we show that the two spike types have different coupling strengths (owing to the unbiased nature of this measure), this would still be an unfair comparison. Because of these considerations, we now limit our conclusions to the fact that many neurons have significant coupling to more than a single CPD for both spike types. To avoid further interpretation of this result, we have removed the distributions of number of coupled frequencies from the relevant figures. We thank the reviewer for pointing out this limitation, and believe that addressing it has led to a more robust interpretation of the results.

4. Does the autocorrelation of spike/bursts contribute to the coupling across temporal scales? ...

We thank the reviewer for the opportunity to clarify our analysis. As the reviewer notices, both tonic spiking and bursting have a short-timescale autocorrelation structure lasting $\sim$ 300 ms (see Figure S2). To specifically investigate coupling that cannot be attributed to this short timescale autocorrelation, we used a permutation test for our statistical analyses. In this approach, the distribution of phase coupling values under the null hypothesis of random spike-CPD phase relationships were determined by partitioning spiking activity into 300 ms bins and shuffling the order of these bins. Consequently, the results presented in this manuscript exclusively capture coupling that surpasses what would simply be expected from the short timescale autocorrelation structure inherent to tonic and burst spikes. In response to this concern, we have further enhanced our description of the permutation method in the main text and Figure 2 caption.

... How are the results influenced by the neurons' spike-generation mechanism? Bursts and (tonic) spikes have distinct firing thresholds and, thus, by definition, occur at distinct time points. Does that contribute to the opposite temporal phase preferences? Bursts occur following periods of hyperpolarization, which must last 100 ms but can be of arbitrary long duration and thus intuitively could show correlation over a range of scales. While an analysis is provided for correlation across scales in the pupil size signal, I have not seen the same for the neural firing. This seems important for the interpretation of the biological interpretation but also to ensure that the results are not due to the spike/burst classification method.

We thank the reviewer for this thoughtful question, but we maintain that several lines of evidence thoroughly rule out the possibility that the burst generation mechanism underlies the phase difference in coupling we observe.

Firstly, even the fastest timescales we are concerned with are an order of magnitude larger than required for the burst generation mechanism. Neurons could theoretically undergo switches in spiking mode many times over these timescales, and our results show that these switches are not random, but have a structure that is tracked by pupil size dynamics. Secondly, the timescale of the burst generation mechanism is constant, but we observe coupling to a diversity of timescales both within single neurons and across the population. Thirdly, a consistent delay between tonic spiking and bursting should result in a linear shift in the relative phase of these two spike types across timescales (a phenomenon thoroughly discussed in our previous reply). Contrary to this, we observe a remarkably consistent phase difference across timescales (Figure 2D, left panel). Indeed, because this phase difference is so consistent, we would expect to find long range temporal correlations between bursting and tonic spiking at multiple time scales, as the reviewer suggests. However, as outlined above, these would not be a direct consequence of the burst generation mechanism.

5. The data in darkness is a great addition, but the infrared wavelength used for eye tracking in darkness is not specified. The mouse retina has low infrared sensitivity, but we have observed sensory responses in the brain in the 'dark-adapted' state at 700-750 nm. The methods should clearly state what measures were taken to ensure complete darkness. ...

We thank the reviewer for appreciating this addition, we also believe it strengthens the manuscript. The LED used for eye tracking operates at a wavelength of 850 nm, and we have now added this important information to the methods section, along with a better description of the recordings in darkness (the stimulus monitor was switched off, and we tried to avoid any illumination by standby lights of equipment etc.). It is important to note that we are not equipped to ensure a "zero-photon" environment, and given that rods and human observers can respond to single photons [10, 13], it is possible that residual changes in retinal illumination, even under our darkness conditions, cause changes in dLGN activity.

Overall, we would like to emphasize that we do not claim that all the modulations we report are solely internally driven. In fact, there is evidence for interesting relationships between pupil size changes, "re-afferent" changes in retinal illumination, the activated retinal circuits, and downstream selectivity of visual cortex neurons [1, 2], which we have now added to our discussion. However, an in-depth investigation of the interaction between ambient illumination and arousal-related modulation of visual circuits is beyond the scope of the present project.

... While the estimated firing rates as a function of pupil size in darkness (S1C1) resemble those in constant illumination (Fig. 1), it does not seem to be the case for Fano factors, which show distinct distributions.

The reviewer is correct in noting that Fano factors are overall slightly higher when the stimulus monitor is on (light median = 1.9 vs. dark median = 1.4; Mann-Whitney-U test: $U = 1.0 \times 10^4$, $p = 2.1 \times 10^{-7}$), consistent with the idea that variability is higher when there is higher baseline activity (light median rate = 7.4 Hz vs. dark median rate = 3.0 Hz; $U = 8.8 \times 10^3$, $p = 6.6 \times 10^{-3}$).

6. The data on movie responses are an interesting addition to the manuscript, but there are issues with the analysis and interpretation of the data. The example raster plot in Fig 5A1 clearly shows how the neuron's response to the visual stimulus toggles between states, showing distinct visual response patterns correlated with changes in pupil size (Fig. 5B1). The marginal plot on the right-hand side, which shows the sum of spike responses over the entire movie, ascribes the trial-to-trial variations in the total number of spikes entirely to noise. However, from the raster plot, the temporal responses to the visual stimulus seem reproducible within a consecutive block of trials. Some of the variations in firing rates over the stimulation epoch may, therefore, not reflect noise but changes in the neurons' visual receptive field (e.g., Aydin et al. 2018, Franke et al. 2022). Are these visual influences taken into consideration in the noise and SNR measurements?

We apologize for the confusing language; the use of the word "noise" was not meant to indicate that we believe these changes in the response pattern and overall rate are random, rather just that we do not yet know what this trial-to-trial variability can be attributed to. Nonetheless, to avoid confusion, we have introduced the term "response variability ratio" (RVR) to describe the observation that trial-to-trial variability dominates stimulus responses.

In agreement with the reviewer, we also believe that these changes result from arousal-related modulations of the neurons' stimulus response properties. Our results later in the figure – almost perfect accuracy for within-state decoding, and a greater decoding penalty across states than across random splits – are evidence that there is arousal-related structure in the variability of stimulus-responses. We have clarified this interpretation in the results

section. Furthermore, we have exchanged the example neuron in Fig. 5 to a more representative one, to avoid creating a false impression regarding the size of the trial-by-trial variability (see also below).

7. The state-dependence of visual receptive fields has implications for the subsequent decoding analyses. Since the pupil state changes the visual responses, how can we be sure that the decoding reflects the dynamic of ongoing activity?

We hope that our response to the point above clarifies that we indeed interpret our results as a reflection of state-dependent changes in the receptive field, and that these considerations extend to the multiple timescales of pupil dynamics that we are concerned with. Because the states we use for within- and across-state decoding are defined by the observed phase coupling of each neuron during the movie stimulus, we believe that the decoding results indeed reflect the ongoing dynamics we observed.

8. The paper has very few illustrations of raw data.

a. Except for Fig. 5A, no burst spike raster plots are shown for the different visual illumination and stimulation conditions (bursts are barely visible in Fig. 5A, and tonic spikes are invisible in Fig. 2B). ...

In response to this concern, in Fig. 5 we now show a different example neuron with more stimulus-driven bursts. With regards to the other conditions (gray screen, sparse noise, and darkness), we would point out that we already show an extensive stretch of raw data in Fig. 1, but that this cannot be shown as a stacked raster plot as there is no definition of a trial. In Fig. 2B our aim was to show several cycles of the example CPD to illustrate the consistency of the coupling, and unfortunately the necessary time window is too large for all individual spikes to be resolved. However, we have increased the size of the panel, and decreased the thickness of the lines representing tonic spikes, such that more individual spikes are now visible.

... Firing rates in Figure 1C1 stem from a model fit of the data. While the fits may be perfect, the data is indirect, and fit quality is not reported. ...

We have followed the reviewer's suggestion and replaced the modeled firing rate in Fig. 1C with raw data (spike counts), and further added measured burst rates (Fig. S3D).

... If the firing events in the neurons show a diversity of phase preferences, then neurons should fire bursts at distinct time points.

While we now show that neurons have some diversity in when they burst as a function of pupil size (Figure S3D), we show that their phase preferences (Figure 2C), especially in relation to tonic spiking (Figure 2D), are strikingly consistent. In Figure 2B it can be seen that this phase preference corresponds to burst spiking clustered at times corresponding to the particular CPD phase.

b. The pupil size data in Fig. S1A is a great addition, but the measurements are normalized to the eye area, which seems unconventional and could prevent comparison to data in previous studies.

In our study we included eye data filmed from a variety of different distances and zoom factors, thus it is not trivial to convert pupil are measured in pixels to mm^2. In the past, we converted pupil sizes by using the main axis of the eye (i.e. a line drawn from the anterior corner to the posterior corner of the eye) as a reference, using published measurements of this axis in mm [12, 14]. However, we found this method to be unreliable, sometimes yielding pupil sizes that were anatomically implausible, perhaps because the actual length of this axis varies between mice. Without a calibration metric, the alternative is to report data in 'arbitrary units' or normalized (e.g. by the maximum size observed in a session). The latter is inappropriate because the maximum pupil size can vary greatly from session-to-session, which prevents a comparison of the range of pupil sizes between sessions/ conditions. Thus, although it may be unconventional, we believe normalizing pupil areas to the total area of the eye visible when the eyelids are fully open is appropriate in this situation. We have added this justification to the methods. To further increase the interpretability of this panel, we no longer plot log-scaled pupil size distributions.

9. The methods are not always properly justified. The use of sparse noise not mentioned in Results. The PoissonGAM modeling is not motivated.

We now explicitly mention the gray screen and sparse noise stimuli at the top of our results section. We use

recordings under these two stimulation conditions as they are iso-luminant and do not induced stimulus-driven pupil dilations. We address the use of the PoissonGAM model in response to 'Line 79 (Details)' below.

10. Statistical analysis. Some p-values are extremely small. This likely is the result from using neurons as experimental unit, instead of animals, which should be avoided if possible. Sample sizes for the statistical tests also seems to be missing. At a minimum the number of mice and units should be specified in the different results sections.

We apologize for the lack of consistent reporting of sample size and have now ensured that sample sizes are present in the main text in every relevant location, as well as in the figure captions. We feel that it is appropriate to use neurons as the experimental unit as there is clear diversity at the neuron level (e.g. Figure 2G), and therefore averaging over neurons would obfuscate our results.

Details:

Line 79: "To characterize the relationship between pupil size and firing rates, we fit a flexible class of modulated Poisson models to spike counts (binned in 250 ms windows)." From the sentence, it is not clear why a model is needed for estimating firing rates. I presume it was to estimate the Fano factor.

The reviewer is correct, to make this specific point it is not necessary to use a model. During the first revision, we decided to implement the PoissonGAM as it is useful for a wider range of analyses. For example, in our previous response to Reviewer 1 we were able to compare the pupil size and its gradient as predictors of spiking activity using the deviance explained by the respective models. The model also allowed us to estimate modulation by pupil size as a smooth function, as opposed to simply binning the data. However, since these considerations are not strictly required for the point we wish to illustrate in the manuscript, we followed the reviewer's wish to see more raw data and now use the empirical firing rates in Fig. 1C and Fig. S1C.

Line 88: "There was also no systematic relationship between the variance of firing rates and the predicted mean (Figure 1C2)" The sentence is weird. Why should we care about the 'predicted' mean?

We hope that this is sufficiently addressed in our response above. We have also decided to remove this result as it is auxiliary to the main point of the figure.

Line 90: "Similar results were obtained in recordings conducted in darkness (Figure S1C), indicating that the overall relationship between pupil size and firing rate observed in our study mainly originates from non-visual factors, such as internal processes related to arousal." Since additional controls are presented later in the manuscript, I suggest toning down the sentence as follows: "Similar results were obtained in recordings conducted in darkness (Figure S1C), suggesting that the overall relationship between pupil size and firing rate originates from non-visual factors."

We thank the reviewer for this suggestion, and have added it to the manuscript.

Line 68: "Pupil size, a marker for internal states such as arousal, was in continuous fluctuation throughout the recordings under constant illumination (see example in Figure 1B, black trace; Figure S1A, gray and pink distributions; $N = 15$ sessions in 10 mice)." This sentence is misleading as some of the recordings are performed upon visual stimulation with sparse visual noise. The use of sparse noise is not mentioned in the Results. This should be specified.

We apologize for the confusion and would like to clarify that the mean luminance during sparse noise stimulation is, for all practical purposes, constant. To avoid any misunderstandings, we have now replaced "constant illumination" with "largely iso-luminant conditions" and have explicitly mentioned in the results section the use of sparse noise experiments. We would also direct the reviewer's attention to Figure R5 and the associated response, where we show that many of the characteristics of phase coupling are nearly identical between conditions.

Line 120: Despite their small contribution to the total spike count Figure S2A1, bursts provide an important extracellular marker for the membrane potential status of thalamic neurons. Bursts additionally play an important role in the determination of rhythmic cortical states [13]. I don't think the words 'important' are necessary. Missing an article before 'determination'.

We have removed both instances, thank you.

Line 139: Across all these time scales, bursts were coupled to a phase opposing that of tonic spiking (Figure 2D, V-test for non-uniform distribution with a mean of π: V = 140.7, p = 0.0). As mentioned above, is the phase difference a property of the coupling or, more simply, a property of the firing rate dynamics?

We believe that this is a property of the coupling, and we hope that we have sufficiently addressed the reviewer's concern in response to point 4 above.

Line 144: suggesting that burst spike may be a more exclusive indicator of the internal state of dLGN neurons. Is this not expected from the membrane potential dependence of bursts? Tonic spikes occur for a range of membrane potentials, whereas bursts require 100 ms of hyperpolarization.

We agree with the reviewer in part, and we have changed this sentence to reflect this. However, it is not wholly expected because it may well be that the membrane potential of dLGN neurons fluctuates between 100 ms of hyper-polarization and depolarization within a single behavioral/ arousal state.

Line 147: indicating that coupling to CPDs is likely driven by changes in internal state rather than visual stimulation. ... 'rather than changes in visual inputs due to pupil size changes' seems more appropriate. Which infrared wavelength was used for eye tracking in general and for eye tracking in darkness?

Thank you for the suggestion, we have updated the sentence accordingly.

Line 150: Together, these results support the notion that dLGN neurons undergo modulations driven by internal factors linked to pupil dynamics across a broad range of temporal scales. Is the result not expected since it takes only 100ms of hyperpolarization to activate T type calcium channels?

We hope that our previous response (point 4) has sufficiently addressed the potential contribution of these short-timescale dynamics.

Line 156: While tonic spiking had a tendency towards multi-scale modulation, with a higher proportion of neurons being coupled to >1 CPDs, bursting was more likely to be modulated at a single temporal scale (Figure 2F; mean number of timescales for tonic spiking: 4.32, mean number of timescales for bursting: 2.49).

We assume that the concern with this sentence is the same as that expressed above (point 3) regarding the difference in statistical power between the two types of events. In accordance with the response above, we have removed this sentence and limit our conclusions to the fact that, for both spike types, significant coupling to multiple CPDs/ timescales could be detected in single neurons.

Line 280: So far, we reported a multi-faceted coupling of dLGN activity to pupil dynamics in the absence of a patterned visual stimulus, but how stable is this coupling in the presence of a rich visual stimulus?

We assume the concern here involves the inclusion of data recorded under sparse noise stimulation until this point in the manuscript. We believe that sparse noise does not qualify as a "patterned" or "rich" and therefore this sentence is appropriate.

Line 225: p-value is missing (truncated). $U = 2.2 \times 10^5$, $p = 2.7 \times 10$

We apologize for this oversight and have corrected it.

Line 505: Were the sparse noise stimuli calibrated to constant luminance? This sentence disregards the sparse noise stimulus in part of the data set.

Yes, the sparse noise was calibrated to constant luminance. We hope that we have sufficiently addressed the reviewer's concern about the sparse noise stimulus above.

The density function of the number of timescales for bursts seems fairly uniform. Different results are seen for bursts and tonic spikes. Which of the two types of activity shows a 'nested' temporal dynamic?

Although we have removed the use of the term in accordance with the reviewer's concern, we would assert that any coupling to more than one timescale qualifies as 'nested' modulations. The modulations at the faster timescale occur *within / on top of* modulations at the longer timescale.

Figure 2B: LEFT, tonic spikes not visible, RIGHT, scale bar missing for polar plot.

As outlined above, we have edited Fig. 2B to make individual tonic spikes more visible. We also now include radial axis grid lines on the polar plots in Fig. 2B providing a scale for probability density.

It is interesting to see that bursts in Fig 5A1 do not seem to be entrained by the visual stimulus. Is that representative of the data set?

As per the response to this concern above, we now provide an example unit that better shows stimulus-driven bursts.

Some the reviewer figures may be worth including in the manuscript as supplementary material (e.g. R1, R2, R3 and R4).

We thank the reviewer's appreciation of these analyses, and have included them where appropriate. We have added original Figure R1 (burst ratios as a function of locomotion) to Figure S4, with the improvement that we now report medians to account for skewed burst ratio distributions. We also believe that original Figure R2 (comparison of the pupil size power spectral density for gray screen and dark recordings) is an appropriate addition, and include this panel, as well as a pupil size PSD comparison between quiescence and locomotion, now in Figure S1.

Original Figure R3 was meant to show that pupil size is a better predictor of dLGN firing rates than the gradient. In addition, it illustrated that the same conclusions that we reach in Figure 1C would also be reached if we had used the gradient instead of pupil size. In accordance with the reviewer's earlier comments we have removed the use of the GAM model from our manuscript and therefore predictions of firing rates by pupil size or its gradient can no longer be made in the original way. Hence, we opted not to include original Figure R3 in the revised version of our manuscript.

We feel that Figure R4 (which compared linear and logarithmic fits to the tonic spike phase shift across timescales) illustrates an inconclusive technical detail. We find it an interesting point of discussion, but have chosen to focus on the biological relevance of our findings as they are of broader appeal. That said, if the reviewer feels strongly about including this panel and the associated discussion, we would of course be happy to do so.

- - - - - - - - - - - - - - - - - - In response to the reviewers' comments about the original submission:

6) In figure 2C, what is p=0.000 mean? Please put $p < 0.001$ or such. To assess the significance of phase coupling, we use permutation tests with 1000 shuffles, allowing us to determine p-values empirically with a precision of three decimal places. Hence, when we report p = 0.000, it means that none of the shuffled samples had a value more extreme than the actual test sample. We apologize for any confusion caused and have now provided clarification in the Methods section.

This notation is not conventional in biology journals. Please use a conventional notation.

We have replaced all such instances with $p < 0.001$ or asterisks denoting significance level.

- - - - - - - - - - - - - - - - - - There are some typos (e.g., missing values and spaces). I have not gone over all of the manuscript in detail but would recommend having a thorough proofread making sure all mistakes are corrected.

We apologize for these errors and have carefully proofread the manuscript to correct these errors.

References

[1] Bouvier G, Senzai Y, Scanziani M (2020) Head movements control the activity of primary visual cortex in a luminance-dependent manner. Neuron 108: 500–511.

[2] Franke K, Willeke KF, Ponder K, Galdamez M, Zhou N, et al. (2022) State-dependent pupil dilation rapidly shifts visual feature selectivity. Nature 610: 128–134.

[3] Huang NE, Shen Z, Long SR, Wu MC, Shih HH, et al. (1998) The empirical mode decomposition and the Hilbert spectrum for nonlinear and non-stationary time series analysis. Proceedings of the Royal Society of London Series A: mathematical, physical and engineering sciences 454: 903–995.

[4] Kutil R (2012) Biased and unbiased estimation of the circular mean resultant length and its variance. Statistics 46: 549–561.

[5] Lopes-dos Santos V, van de Ven GM, Morley A, Trouche S, Campo-Urriza N, et al. (2018) Parsing hippocampal theta oscillations by nested spectral components during spatial exploration and memory-guided behavior. Neuron 100: 940–952.

[6] Niell CM, Stryker MP (2010) Modulation of visual responses by behavioral state in mouse visual cortex. Neuron 65: 472–479.

[7] Piu P, Serchi V, Rosini F, Rufa A (2019) A cross-recurrence analysis of the pupil size fluctuations in steady scotopic conditions. Frontiers in Neuroscience 13: 407.

[8] Quinn AJ, Lopes-dos Santos V, Huang N, Liang WK, Juan CH, et al. (2021) Within-cycle instantaneous frequency profiles report oscillatory waveform dynamics. Journal of Neurophysiology 126: 1190–1208.

[9] Reimer J, Froudarakis E, Cadwell CR, Yatsenko D, Denfield GH, et al. (2014) Pupil fluctuations track fast switching of cortical states during quiet wakefulness. Neuron 84: 355–362.

[10] Rieke F, Baylor DA (1998) Single-photon detection by rod cells of the retina. Reviews of Modern Physics 70: 1027.

[11] Saleem AB, Ayaz A, Jeffery KJ, Harris KD, Carandini M (2013) Integration of visual motion and locomotion in mouse visual cortex. Nature Neuroscience 16: 1864–9.

[12] Schmucker C, Schaeffel F (2004) A paraxial schematic eye model for the growing C57BL/6 mouse. Vision Research 44: 1857–1867.

[13] Tinsley JN, Molodtsov MI, Prevedel R, Wartmann D, Espigulé-Pons J, et al. (2016) Direct detection of a single photon by humans. Nature Communications 7: 12172.

[14] Wisard J, Chrenek MA, Wright C, Dalal N, Pardue MT, et al. (2010) Non-contact measurement of linear external dimensions of the mouse eye. Journal of Neuroscience Methods 187: 156–166.
