# Supplementary material for: Spiking activity in the visual thalamus is coupled to pupil dynamics across temporal scales
Source: PLoS Biol. 2024 May 14;22(5):e3002614. doi: 10.1371/journal.pbio.3002614 (PMC11093384; doi:10.1371/journal.pbio.3002614)
Supplement: Attachment — Submitted filename: Crombie_et_al_rebuttal.pdf [file pbio.3002614.s010.pdf]

Dear Dr. Smith, dear editorial team, dear reviewers,

We would like to thank you for a further opportunity to revise our manuscript "Spiking activity in the visual thalamus is coupled to pupil dynamics across temporal scales", and express our sincere gratitude to the reviewers for their time the took for our manuscript and their helpful feedback. We appreciate that some of the analyses presented in our manuscript are quite involved, but we believe that the reviewers' comments in this round of revisions have helped to highlight the biological significance of our findings, to make the manuscript easier to read and to further broaden its appeal.

Here is an overview of the major changes to the manuscript:

1) We fully agree with Reviewer #3 that there is no contradiction between using pupil size *per se* as an indicator of behavioural state and our approach. We would like to emphasize that it was never our intention to diminish the usefulness of pupil size *per se* as an indicator of internal state. In fact, the focus on pupil size was introduced in previous round of revisions with the sole goal of providing more context and a better motivation for our analyses. To prevent that our manuscript will be perceived as downplaying the usefulness of pupil size *per se*, we re-arranged figure panels highlighting the explanatory power of pupil size and now present them in the main text (e.g., Figure 1C). In addition, we have made substantial revisions to the introduction, results, and discussion to ensure that we view pupil size and its dynamics as complementary indicators for internal modulations.

2) As suggested by both reviewers, we have included some of the figure panels and associated explanations from the previous response to reviewers in the manuscript.

3) Throughout the manuscript, we have followed the suggestions of Reviewer #4 to remove methodological detail from the manuscript, better introduce technical terms when they are required, and better emphasize the biological significance of our findings.

In addition to the above, we have implemented the remaining suggestions and addressed the reviewers concerns, detailed in the point-by-point response to the comments below.

We have provided a document tracking all of the changes to manuscript, which are highlighted in red. We hope that we could implement the changes as intended by the reviewers.

Reviewer #3: I'd like to thank the authors for performing additional analysis to address my comments and concerns. I suggest to include Reviewer Figures R1 C-E and R4 in the manuscript as supplementary materials for readers to assess the data variability and conclusions.

We thank the reviewer for this suggestion, and have included Figures R1 C-E in Figure S3.

We enthusiastically agree with the reviewer on the inclusion of pupil size tuning data for firing rates, and also for burst rates – a previous reviewer figure that we have now placed in an emphasis position in the manuscript (Figure 1C2; see also our response to point 1). However, we strongly prefer not to include the pupil size derivative as shown in Figure R4 into the manuscript for the following reasons: In lieu of heavily smoothing the data, or averaging over trials, we would argue that the derivative is over-representative of noise in the signal. Even if one chooses to use the smoothed/ averaged derivative, it has been argued elsewhere that this is redundant as the size derivative and size itself are correlated with a delay (e.g. McGinley et al., 2015, Neuron). We also feel that an operation like smoothing is similar to considering dynamics at a limited range of timescales (i.e. slower timescales, dependent on the width of smoothing kernel used). In this respect, we are convinced that our analysis in the remainder of the paper is a more principled approach to extract the same information.

My other comments below mostly involve text changes.

1. To motivate the multi-timescale arousal modulation of dLGN spiking activity, the authors highlighted that 'pupil size provided only limited information about firing rate: variance of firing rates within pupil size bins was high and in fact often larger than the mean (Figure 1C2; median Fano factor = 2.0' (Line: 86-87). However, the phase coupling strengths across a broad range of temporal scales were all notably small (with an R^2 of 0.02 for burst firing and even smaller for tonic firing, within a theoretical range of R^2 from 0 to 1). Moreover, the variance of the phase

coupling may also be large. If Fano factors were computed for bursts and tonic spikes at each phase, the resulting Fano factors would likely be substantial, just as what was shown in Figure 1C. It remains challenging to determine whether pupil size analysis already captured the majority of the modulation while multi-timescale analysis provided supplementary information, or vice versa. Therefore, it is essential not to diminish the significance of much simpler pupil size analysis (including analysis of both the absolute value of pupil sizes and pupil size gradients) in relation to dLGN spiking activity. In fact, the multi-scale pupil dynamic analysis may help to more closely capture the pupil size and pupil size gradient on both local and global temporal scales.

We completely agree that there is great value in the use of using pupil size *per se*, and never intended to directly compare pupil size versus its component dynamics. We also agree with the reviewer's final sentence, regarding how our findings can be contextualized in relation to pupil size and its derivative. We would not advocate for trying to interpret absolute values of measures such as bias-corrected R-squared. Nonetheless, the reviewer's impression that we are diminishing the importance of a measure such as pupil size prompted the following changes:

Firstly, we now further emphasize the findings in Figure 1C related to pupil size in the main text. Specifically, we have moved figure panels showing the relationship of burst rates to pupil size from a supplementary panel to Figure 1C and highlight that the majority of neurons in dLGN are modulated by pupil size, and that a sizeable fraction have a monotonic relationship to pupil size. Secondly, we have moved the panel with the analysis of Fano factors to supplementary Figure SC3 and have changed it to a more compact representation. We have also re-positioned the description of this result in the main text and now only use it to transition to our new analysis, without implying inadequacy for pupil size as a predictor. Finally, we have carefully re-worded our introduction, Figure 3 results section, and discussion to make sure we are not unfairly downplaying the usefulness of pupil size *per se*.

2. Line 218-219: I am not convinced the conclusion that 'locomotion-dependent modulations appear unrelated to the phase coupling reported above' can be drawn based on the observation that 'significant coupling for tonic spikes was equally likely regardless of whether the component was correlated to locomotion or not', as for those that had correlated locomotion and CPD components, the locomotion can still be related to phase coupling. We completely agree with the reviewer and apologize for the poorly-worded conclusion. We have revised this sentence as follows to better reflect the actual results described:

"Thus, while locomotion can drive changes in spiking activity, and locomotion speed is partially reflected in certain CPDs, these relationships do not appear to be the sole cause of coupling to pupil dynamics."

3. Line 210: Figure S1B4 does not exist.

We believe this must be a misunderstanding, as the referenced figure panel is present.

Reviewer #4: Most of my comments and concerns have been addressed satisfactorily. This is an extensive revision which has greatly improved the statistical analysis and demonstrate the robustness of the results. I have a few remaining minor concerns about the writing and interpretation of the data

We thank the reviewer for the positive evaluation of our revision.

General comments

1. In comment 1, Reviewer 1 asked about the reliability of the phase coupling measurement in individual neurons. In their response, the authors show convincingly that similar phase and frequency preferences are observed at the population distribution level across subsamples of the data but I could not find the data for the individual neurons' across subsamples. This is important because there is a repeated claim of diversity of coupling within the population, but because the results vary across sessions and mice this can only be shown by examining the coupling results for simultaneously recorded neurons from a same animal and experimental session across data subsamples. If I am not mistaken, no such data is presented.

Thank you for expressing this concern. We would point out that all analyses in the previous reviewer figure (Figure R1 C-D, reproduced below) already involve a within-neuron comparison across sub-samples as requested, and we apologize if this was not clear. Since the panels are now included in the supplements, we have written the figure caption to ensure that it is clear that this is a within-unit comparison (see caption below). While the

distributions shown are indeed pooled across recordings and mice, the proportions of neurons following the pattern of similar phase and frequency preference across sub-samples is overwhelming (> 80%). Therefore, despite diversity within and across sessions in neurons' timescale preferences, the pattern of results we wanted to emphasize must be present across the large majority of the recordings.

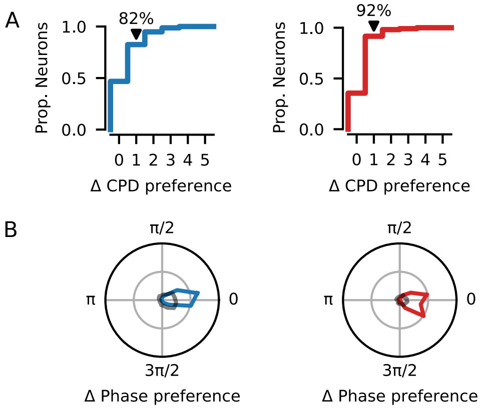

Figure (A, B) Preferred timescale and phase tends to be stable across data sub-samples (*blue*: tonic spikes, *red*: bursts). The phase coupling of individual neurons was compared across each of two interleaved partitions of the data (see Methods). The change in the CPD to which a neuron is most strongly coupled is shown in (A) for all neurons, 0 indicates that the strongest CPD remained unchanged, 1 indicates that the strongest CPD shifted to a CPD adjacent in characteristic timescale. The overwhelming majority of neurons either retain the same preferred CPD or switch to the next closest timescale. The change in preferred phase for each neuron-CPD pair is shown in (B) for pairs in which the coupling was significant in both sub-samples (*colored distribution*) and in which coupling did not reach significance in one or both of the sub-samples (*gray distributions*). For both tonic spikes and bursts, the change in phase preference was $< 90°$ for $> 90\%$ of neurons.

2. In their response to my comments, the authors claim that the differential coupling of spikes and bursts cannot be explained by the neurons' intrinsic properties such as the spike generation mechanism. I don't quite follow the argumentation. However, as the new example cell in Figure 5 makes abundantly clear, bursts in the LGN strictly occur during periods of low level of single spike activity which, intuitively, could explain the oppositive phase preferences of bursts and single spikes. This hypothesis that could be addressed using computer simulations. If not, the authors should address the topic in Discussion and explain how this intuition is incorrect.

The observation made by the reviewer is, in fact, direct evidence arguing against the involvement of the burst generation mechanism in the opposing phase preferences of tonic spikes and bursts. We now comment on this in the results section, the argumentation is as follows: Our analysis in Figure 5C2 shows that it is particularly for fast timescales (i.e. those observed by the reviewer in the example neuron) where the tonic-burst phase relationship breaks down during stimulus viewing. The fact that tonic spikes and bursts do not *always* have opposing phases preferences is strong empirical evidence that the intrinsic properties of dLGN neurons are not sufficient to produce this result.

Aside from this empirical evidence, there are several *a priori* arguments against the direct involvement of the burst generation mechanism in our findings, in brief:

- T-type calcium channel de-inactivation dynamics only guarantee a temporal offset of approximately 100 ms, our results show consistent relationships at much longer timescales, up to several minutes
- In Figure 5A bursts generally occur during periods of low tonic spiking, but high tonic spiking can occur immediately after a burst, which would give rise to similar, rather than opposite, phase preferences
- The timescale of the burst generation mechanism is constant, but we show opposing phase relationships for diverse timescales within and between neurons
- A constant temporal delay between bursting and tonic spikes should result in a linear relative phase shift between the two events across timescales, but we show that the relative phase is constant across timescales
- Cell-intrinsic biophysical mechanisms may explain a temporal relationship between the two spike types, but

cannot alone explain why this should be aligned with pupil size dynamics and in a manner that is coordinated across the population of neurons, implicating some other cause such as modulatory influences, common inputs, network effects, etc.

3. Some additional points or questions addressed in the response to reviewers, seem not to have been considered or integrated in the manuscript revision. I would consider integrating these data and analyses to the manuscript, at least in the Discussion or as supplementary material.

We agree that some of the responses included in the reviewer replies, but not in the manuscript, are of value. To this end we have followed the suggestion, also expressed by reviewer #3 above, to include some of the panels from the previous reply in the manuscript. However, we also feel that many of these replies addressed very technical points that, while important, may not be of interest to a general biological audience. We are aware, as the reviewer voices below, that the manuscript may already contain too much technical detail.

4. Despite comments from the reviewers, the Results and the figure captions still contain jargon (e.g. '(characteristic) time scales', 'characteristic frequency', 'phase synchronization', 'phase-coupling', 'phase-coupling framework'). These terms are introduced without explanation or in way that is not adapted to the broad readership of the journal. The authors should screen for such words, add definitions in place (not buried in the Methods) or replace them with concrete explanations or simpler terms.

Thank you for expressing this concern. By the nature of our study, we feel that it is difficult to completely eliminate this terminology. Nonetheless, we have removed these terms where possible, and replaced with descriptions that more explicitly emphasize the relevance of the concept for our later results. For example, to remove "phase synchronization", we have revised the text as follows:

Original: "Importantly, the set of components extracted from a single pupil trace exhibited only limited periods of phase synchronization (Figure S1D), indicating that dynamics at different timescales were largely independent of each other.

Revised: "Importantly, the set of CPDs extracted from a single pupil recording progress through dilation-contraction cycles largely independently from each other (Figure S1D; see Methods), indicating that CPDs capture distinct aspects of pupil size dynamics."

For other cases where we believe it is important to maintain the terminology ("characteristic timescale", "phase coupling"), we have focused on making the biological significance explicit, rather than providing a technical definition.

Original: "Taking the circular mean angle of these collected phases allowed us to determine the preferred phase of tonic spiking/ bursting. To quantify the strength of phase coupling, we used the bias-corrected squared circular mean length [30], which allowed comparisons between neurons with different firing rates and between bursting and tonic spiking. Finally, to evaluate the significance of the phase coupling, we compared the observed coupling strength to a null-distribution of coupling strengths obtained from shuffled surrogate spike trains. This procedure specifically accounts for the presence of short timescale spike patterns ($\leq$300 ms; Figure S2B; see Methods) that typically inflate coupling strengths."

Revised: "The preferred phase of each spike type indicates whether they mainly occur during pupil dilation ($-\pi$ to 0) or contraction (0 to π) as captured by the CPD. Meanwhile, the coupling strength indicates the degree to which spikes and bursts adhere to the preferred phase and was computed with a bias-corrected metric that allows comparison between neurons with different rates of tonic spiking and bursting (see Methods; Figure S2A). The statistical significance of the coupling was assessed using a permutation test that accounts for short-timescale spiking patterns that typically inflate coupling strength metrics (see Methods; Figure S2B)."

We have not made changes to the figure captions, as here it is important to maintain both brevity and precision, which is the intended purpose of the technical language.

5. Along the same lines, the wording of the findings and conclusions in the abstract and in the manuscript are phrased in a very technical language that is inaccessible to 99% of biologists. For a journal like PLOS Biology, the findings should be conveyed in more simple language (e.g. "single spikes and bursts of action potentials in the visual

thalamus preferentially occur at distinct, approximately opposite phases of the pupil behavior indicating ...") to be accessible to biologists from a different field (e.g. molecular or developmental biology).

As outlined above, we have taken care to use less technical language where possible, especially in the abstract, where we thank the reviewer for the specific suggestions.

6. With the revisions, the Results has become somewhat bloated with methodological sentences. I recommend moving these sentences to Methods to improve readability.

We thank the reviewer for pointing this out. We have taken care to do so where possible (see response to point 4 above).

7. It is good practice to include in Discussion a paragraph on limitations of the study. This seems to be missing.

We completely agree that accurately representing the limitations is important, and apologize if these limitations were not clear previously. We have revised the discussion to make these instances more clear, but we have chosen to do so across separate paragraphs, incorporating the points as they arise. Here are the most prominent examples (some new, some old):

- "It is important to note that the components we recovered here do not necessarily map directly onto the time-courses of independent internal processes or behavioral sequences."
- "While providing a quantitative account of dLGN modulations linked to multi-scale pupil dynamics, locomotion, and saccades, a limitation of our study is that it has not addressed these modulations on a mechanistic level."
- "Changes in retinal irradiation linked to pupil size can shift color selectivity in the visual system [39], and there is evidence for interactions between luminance and behavioral modulation [67] that warrants further exploration. Future studies directly tracking and manipulating the activity of these modulatory systems in the thalamus promise to yield further insight into the relationship between internal states and feed-forward sensory signals across diverse time scales."
- "It remains to be seen whether spatio-temporal receptive fields change across phases of pupil dynamics, and whether these changes can be attributed to membrane hyperpolarization and bursting."
- "Moving forward, considering the specific functional role of a given neuron, along with its temporal structure of modulation, will lead to a better understanding of dynamic stimulus processing in the visual system."
- "While our study has expanded the use of the pupil signal to reveal arousal-related modulations to multiple temporal scales, recent work has emphasized that a larger array of behaviors may also help to better explain activity in sensory systems [19, 86]."

Details:

L64 First sentence does not seem to encompass the overall goal of the study.

Thank you for pointing this out, we have revised the opening sentence, and, indeed, the opening paragraph to better reflect the goals of he study.

L67 It would make sense to mention that pupil size variations also change retinal illumination, which can also affect the dynamics of visual responses.

We agree that the point is important, so we now mention it when introducing the results from recordings in darkness, and have edited the discussion to clarify our thoughts on this topic, reproduced below:

"Furthermore, although our analyses in darkness control for changes in retinal illumination linked to pupil size *per se*, there are known effects of pupil size on feed-forward signaling. Changes in retinal irradiation linked to pupil size can shift color selectivity in the visual system [39], and there is evidence for interactions between luminance and behavioral modulation [67] that warrants further exploration."

L93 The firing events in 2 seem to occur in absence of pupil fluctuations. Is this an example of a non-pupil driving firing event or are their fluctuations that are too small to be resolved?

Thank you for noticing this, we have chosen to emphasize this example because we think it illustrates one of the points we try to make throughout the manuscript. Although this event does not directly relate to pupil dilation, it clearly relates to a dilating phase of the third component of pupil dynamics. We would thus emphasize our interpretation that the raw pupil size dynamics are the result of many underlying component influences, only some of which may be relevant to a given neuron, thus the activity of the neuron may be related to the component but not the raw signal.

L94 It would make sense to point out how these different types of firing events could reflect different types of modulations.

Agreed, this is exactly our interpretation as well. We would like to direct the reviewer's attention to our revised discussion, where we outline the multitude of influences – neuromodulatory, behavioural, and cognitive – that might underlie the fast versus slower modulations we observed. In particular, we have added descriptions of brainstem nuclei with influence in the dLGN that are linked to locomotion and eye movements, factors that we show to also have an influence on dLGN activity, but whose relationship to pupil size dynamics remains unclear.

Figure 1: panel D1 is difficult to parse. The repeated gray lines that overlap with the colored lines do not really help to understand the decomposition. I understand that the authors would like to highlight the correlation but what is salient are the deviations. I would rather plot gray and colored lines separately showing how colored lines represent a decomposition of the gray line by putting them next to each other.

We address this point below (point L105).

Figure 1: D2 caption uses both the terms "characteristic frequency" and "characteristic timescale". Only the latter is defined in Methods. Pick one and use throughout the manuscript. Furthermore, these terms are not introduced in Results. Either include in Results or discard.

Thank you for catching this, we replaced the instance of characteristic "frequency" in the figure caption, and have added a sentence to the main text to more explicitly introduce "characteristic timescale".

L97: "beyond the size" what the authors means is "absolute, instantaneous size".

The reviewer has interpreted correctly, but we do not believe it is appropriate to add the qualifiers, which both come with their own problems in interpretation. For example, our statement would still be correct if pupil size was averaged in a small window around the timepoint, and the size cannot be absolute in our study as it is expressed as a function of the size of the eye. Although we note that it is common to simply use "pupil size" to refer to this quantity, throughout the text (in the passage the reviewer is quoting from, for example), we employ the qualifier *per se* to distinguish between pupil size and its dynamics. We hope this helps clarify any misunderstanding.

L104: missing reference for "empirical mode decomposition".

Thank you, we have added the reference ([92]) where relevant in the main text and methods section.

L105: The text "capturing its underlying dynamics" does not seem to be adequately supported...

Please see our response below.

...The motivation for choosing this specific decomposition is not explained. No reasoning explaining how this decomposition is more appropriate than others is presented (e.g. Fourier decomposition, wavelet transform, etc)...

We apologize for this lack of clarity, we now elaborate on our choice of decomposition in the methods section.

"In contrast to the Fourier or wavelet transforms, EMD extracts these components without pre-defined filters, and can capture nonstationarities present in biological signals with individual frequency- and amplitude-modulated components [92]."

...The time sequences in Figure 1D1 seem to suggest otherwise as to the eye only one of the components seems strongly correlated with pupil size. Furthermore, the absolute measure of explained variance and absolute correlations are not provided.

We would like to emphasize that components extracted by a decomposition do not need to strongly correlate with the original signal, otherwise there would be no point in doing the decomposition (this is true also for other decomposition methods like the Fourier transform). The degree to which a given component contributes to the original signal is given by the power, which is indicated by the labels beside each example component, and for every component in Figure 1D3. We decided to overlay each component with the original signal in Figure 1D1 so that the reader can see which aspects each component captures. In contrast to the reviewer's statement, we strongly feel that there are many instances, distributed across all components, where the relationship between the component and the original signal are clear. We would point out that these relationships might only occur for limited periods of time, when the dynamics are actually present in the original signal, highlighting the benefit of our chosen decomposition method which captures such nonstationarity (see above). However, for the reader to make these observations the overlay of the component with the original signal is required.

We hope this response also sufficiently addresses the reviewer's concerns from above regarding the overlay gray lines in Figure 1D1, and the concern about capturing the "underlying dynamics".

Figure 1D3 the caption is not clear. What are the gray dots? A box plot cannot show 'a distribution'.

We have re-worded the figure caption, and clarified that the box plot only shows the range and IQR of the distribution. We hope these changes help to enhance the clarity.

Line 108-110: Figure 1D3 is cited to support the claim that components were found/extracted but no data on effect size and significance levels is shown. Some of the datapoints lie on the abscissa. Presumably these data points are not significant.

Thank you for raising this concern, which has prompted a revision of how this panel is described to try to make a couple points more clear. Firstly, across many timescales there are components that have relatively high power values, indicating that the pupil size signal truly is multi-component. Secondly, the box plot showing diversity in the characteristic timescale of the highest power component is meant to illustrate that pupil dynamics are diverse across recordings. In addition to clarifying the figure caption, we have added a label to the figure that we hope makes this more obvious.

Low power components are indeed present, but "significance" is not an attribute that can be assigned in the absence of a null hypothesis about which aspects of the signal are meaningful. Throwing away components using an arbitrary power threshold would bias our analysis, as even low power components have neurons that are coupled to them, indicating that they are "significant" from the perspective of dLGN activity modulations.

Line 155-156: The statistical testing for this paragraph is not clear. Which threshold was used to test the significance of coupling modulations at multiple time scales. Were the results corrected for multiple comparisons? Perhaps the answer lies in the following sentences but the statistical tests and sample sizes supporting these statements are not clear.

We have made an edit to the text to clarify that "coupling to multiple timescales" simply means that there were multiple CPDs for which the coupling reached our threshold for significance. The threshold used was $\alpha \leq 0.05$, and not corrected for multiple comparisons. For the reviewer, we have repeated the analysis applying the Bonferroni correction ($alpha$ / N, where N is the number of CPDs per neuron, typically 6), and obtained the same conclusion (tonic spiking mean = 3.2 CPDs per neuron, uncorrected = 3.8; bursting mean = 1.4 CPDs per neuron, uncorrected = 2.1). However, we do believe this correction to be overly-conservative for the following reasons. Although CPDs are derived from the same original signal, our analysis in Figure 1D shows that their phase progression is largely independent. Furthermore, for the conclusion that single neurons are coupled to multiple components, we have already identified and removed all phase combinations in which a CPDs are themselves coupled. Therefore, we maintain that our statistical testing procedure, which already accounts for the statistical dependence between variables involved in the repeated comparison, is sufficiently stringent to support our conclusions.

L168-171: Speculation. Does not belong to Results. The source of the variability across mice or recording sessions

is not directly addressed.

This must be a misunderstanding, and we apologize for the confusion. This statement is derived directly from a result presented in the supplementary Figure S3C1 that was references with the sentence. We now additionally include the results of this correlation in the main text to clarify that this statement is not speculative.

L305: Variability

Thank you for catching this.
